# Impact of macronutrient supplements on later growth of children born preterm or small for gestational age: A systematic review and meta-analysis of randomised and quasirandomised controlled trials

**DOI:** 10.1371/journal.pmed.1003122

**Published:** 2020-05-26

**Authors:** Luling Lin, Emma Amissah, Gregory D. Gamble, Caroline A. Crowther, Jane E. Harding

**Affiliations:** Liggins Institute, University of Auckland, Auckland, New Zealand; London School of Hygiene and Tropical Medicine, UNITED KINGDOM

## Abstract

**Background:**

Nutritional supplements may improve short-term growth of infants born small (preterm or small for gestational age), but there are few data on long-term effects and concerns that body composition may be adversely affected. Effects also may differ between girls and boys. Our systematic review and meta-analysis assessed the effects of macronutrient supplements for infants born small on later growth.

**Methods and findings:**

We searched OvidMedline, Embase, Cochrane CENTRAL, and Cochrane Database of Systematic Reviews from inception to January 30, 2020, and controlled-trials.com, clinicaltrials.gov, and anzctr.org.au on January 30, 2020. Randomised or quasirandomised trials were included if the intention was to increase macronutrient intake to improve growth or development of infants born small and growth was assessed after discharge. Primary outcome was body mass index (BMI) in childhood. Data were pooled using random-effect models. Outcomes were evaluated in toddlers (< 3 years), childhood (3 to 8 years), adolescence (9 to 18 years), and adulthood (>18 years). Forty randomised and 2 quasirandomised trials of variable methodological quality with 4,352 infants were included. Supplementation did not alter BMI in childhood (7 trials, 1,136 children; mean difference [MD] −0.10 kg/m^2^, [95% confidence interval (CI) −0.37 to 0.16], *p* = 0.45). In toddlers, supplementation increased weight (31 trials, 2,924 toddlers; MD 0.16 kg, [0.01 to 0.30], *p* = 0.03) and length/height (30 trials, 2,889 toddlers; MD 0.44 cm, [0.10 to 0.77], *p* = 0.01), but not head circumference (29 trials, 2,797 toddlers; MD 0.15 cm, [−0.03 to 0.33], *p* = 0.10). In childhood, there were no significant differences between groups in height (7 trials, 1,136 children; MD 0.22 cm, [−0.48 to 0.92], *p* = 0.54) or lean mass (3 trials, 354 children; MD −0.07 kg, [−0.98 to 0.85], *p* = 0.88), although supplemented children appeared to have higher fat mass (2 trials, 201 children; MD 0.79 kg, [0.19 to 1.38], *p* = 0.01). In adolescence, there were no significant differences between groups in BMI (2 trials, 216 adolescents; MD −0.48 kg/m^2^, [−2.05 to 1.08], *p* = 0.60), height (2 trials, 216 adolescents; MD −0.55 cm, [−2.95 to 1.86], *p* = 0.65), or fat mass (2 trials, 216 adolescents; MD −1.3 5 kg, [−5.76 to 3.06], *p* = 0.55). In adulthood, there also were no significant differences between groups in weight z-score (2 trials, 199 adults; MD −0.11, [−0.72 to 0.50], *p* = 0.73) and height z-score (2 trials, 199 adults; MD −0.07, [−0.36 to 0.22], *p* = 0.62). In subgroup analysis, supplementation was associated with increased length/height in toddler boys (2 trials, 173 boys; MD 1.66 cm, [0.75 to 2.58], *p* = 0.0003), but not girls (2 trials, 159 girls; MD 0.15 cm, [−0.71 to 1.01], *p* = 0.74). Limitations include considerable unexplained heterogeneity, low to very low quality of evidence, and possible bias due to low or unbalanced followup.

**Conclusions:**

In this systematic review and meta-analysis, we found no evidence that early macronutrient supplementation for infants born small altered BMI in childhood. Although supplements appeared to increase weight and length in toddlers, effects were inconsistent and unlikely to be clinically significant. Limited data suggested that supplementation increased fat mass in childhood, but these effects did not persist in later life.

**PROSPERO registration**: CRD42019126918.

## Abstract 2

Jane Harding and co-workers investigate possible effects on growth and weight in later life of macronutrient supplementation for children born small or preterm.

## Author summary

**Why was this study done?:**

- Preterm and small-for-gestational-age infants are at increased risk of poor growth.
- Nutritional supplements are often given to these infants to improve short-term growth, but observational studies suggest that this may lead to increased risk of later overweight/obesity.
- There are few data on long-term effects, and it is possible that they may be different in girls and boys.

**What did the researchers do and find?:**

- We undertook a systematic review and meta-analysis of 40 randomised clinical trials and 2 quasirandomised trial of nutritional supplements involving 4,352 infants born preterm or small for gestational age.
- We found that early supplements given to infants born small did not alter BMI in childhood but may slightly increase weight and length in toddlers and fat mass in childhood.
- None of the effects persisted after early childhood, although data are limited and quality of evidence is low.

**What do these findings mean?:**

- The available evidence suggests that early nutritional supplements for infants born preterm or small do not alter BMI in childhood but also have little effect on growth.
- Despite large numbers of trials involving thousands of infants, there is still limited evidence about the benefits and risks of early nutritional supplements after early childhood.

## Introduction

Infants born preterm or small for gestational age (SGA) are at increased risk of poor growth, delayed development, and disability [1–4]. Preterm infants accumulate significant protein and energy deficits during early life [5], which can cause a substantial postnatal growth failure. Approximately 80% of preterm infants [6] and 85%–90% of SGA infants [7] attain catch-up growth after initial growth failure, although incomplete catch-up growth is common [6], and these children often remain considerably smaller than their full-term peers during childhood, adolescence, and adulthood [5,6,8,9].

Further, infants born small often have significantly lower bone mineral content than those born at term. Approximately 80% of total body bone mineral content of the fetus is acquired in the last trimester of pregnancy, so inadequate mineral transfer from the placenta before birth will lead to reduced bone mineral content in preterm infants [10–12]. The prevalence of metabolic bone disease is inversely associated with birth weight and gestational age [11,12] and may increase the risk of neonatal rickets, childhood fracture, and poor growth in childhood [12].

Providing preterm and SGA infants with enhanced nutrition in early life is associated with improved short-term growth [13–18]. However, observational studies suggest there may be an increased risk of later overweight/obesity after the period of rapid early weight gain [19–21] and that this may contribute to increased risk of later metabolic disease, diabetes, and heart disease in those born small [22,23]. There also is limited evidence that these effects may differ in boys and girls [24]. Three previous systematic reviews have compared the effect of supplemented versus unsupplemented formula started after hospital discharge and fortified versus unfortified breastmilk started in hospital or after hospital discharge [17,25,26]. None of these reviews reported growth outcomes after 18 months of age, and none assessed potential sex-specific effects.

The aims of this systematic review and meta-analysis were to assess the effects of macronutrient supplements in nutrition of preterm and SGA infants on later growth and bone development after hospital discharge and to assess whether these effects differ in girls and boys.

## Methods

This study is reported as per the Preferred Reporting Items for Systematic Reviews and Meta-Analyses (PRISMA) guideline (S1 Checklist) and registered prospectively in PROSPERO (registration number CRD42019126918) (S1 Protocol).

Ethics approval was not required for this analysis of published data.

### Search strategy and selection criteria

We searched OvidMedline, Embase, Cochrane Library Central Registry of Controlled Trials, and Cochrane Database of Systematic Reviews from inception to January 30, 2020. We also searched for eligible ongoing trials in Current Controlled Trials (www.controlled-trials.com), Clinical Trials (www.clinicaltrials.gov), and the Australian and New Zealand Clinical Trials Registry (www.anzctr.org.au) on January 30, 2020. Conference abstracts were included if they provided usable data. Search strategy and search terms are in S1 Table.

Inclusion criteria were as follows: (1) randomised controlled trials (RCTs) and quasi-RCTs without restrictions on date of publication or language; (2) infants born preterm (<37 weeks’ gestation) or small (birth weight <2.5 kg or <10th percentile); (3) the intervention was intended to increase intake of one or more macronutrients (protein, carbohydrate, fat, energy, or protein/energy ratio) with the primary aim of improving growth or development (interventions could be enteral, parenteral, or both; commence any time during initial hospitalisation after birth or after discharge; and must have been provided for ≥1 week); and (4) reported any of the prespecified outcomes assessed after term equivalent age (>37 weeks’ postmenstrual age) or after discharge from hospital after birth.

Studies that reported comparisons between unsupplemented and supplemented nutrition with parenteral supplements, human breast milk supplements, formula milk, or other macronutrients were eligible for inclusion. We excluded trials comparing the timing of the introduction of nutrition (early versus delayed feeding), macronutrients of different composition (e.g., different types of lipids or proteins), variations in intakes of micronutrients (including sodium, potassium, calcium, phosphorous, vitamins, other minerals, amino acids, fatty acids), or focussed on gastrointestinal development.

The primary outcome was body mass index (BMI) in childhood.

Secondary outcomes were (1) growth assessments: weight (absolute and z-scores), length or height (absolute and z-scores), head circumference (absolute and z-scores), ponderal index, and body composition (fat mass, lean mass, measured by bioimpedance or DEXA or skin fold thickness or other method); (2) bone development: bone mineral content, volumetric bone mineral density, and fractures; (3) nutrition: feeding tolerance, intake (protein, energy), appetite, breastfeeding and duration; (4) death: neonatal or later death, up to the time of followup and cause of death; (5) quality of life; (6) general health and use of healthcare resources; (7) adverse events; and (8) cost. When data were available, the outcomes were evaluated in toddlers (<3 years), childhood (3 to 8 years), adolescence (9 to 18 years), and adulthood (>18 years). However, there were no data for ponderal index, fractures, nutrition, death, quality of life, general health and use of healthcare resources, adverse events, and cost.

### Data collection and analysis

Two reviewers (LL and EA) independently screened titles and abstracts of all records identified, assessed potentially eligible full-text articles for inclusion, extracted data into a template data extraction form, and assessed the risk of bias for included studies using Cochrane criteria [27]. Discrepancies were resolved by discussion or with a third author (JH).

We assessed risk of bias for each key outcome using the Grading of Recommendations Assessment, Development and Evaluation (GRADE) approach [28] and created ‘Summary of findings’ tables using GRADEpro Guideline Development Tool [29]. If a trial reported the same outcomes measured at different time points in childhood or beyond (>3 years), we chose the age group with the most data for assessment of the quality of evidence. We assessed quality of evidence for the following outcomes (GRADE outcomes): BMI in childhood, weight in toddlers, head circumference in toddlers, length or height in toddlers, height at >3 years, lean mass at >3 years, and fat mass at >3 years.

### Statistical analysis

We undertook meta-analyses using RevMan 5.3 [30] using random-effects models and calculated relative risks (RRs) and mean differences (MDs) with 95% confidence intervals (CIs). For measures of bone mineralisation, we calculated standardised MDs (SMDs) with 95% CIs because of different methods of measurement. *P*-values < 0.05 denoted statistical significance. All tests were 2-tailed with no adjustment for multiple comparisons. We calculated *I*^2^ and *χ*^2^ tests to determine statistical heterogeneity, with *I*^2^ > 50% and *P* < 0.10 considered significant heterogeneity. We assessed bias due to small study effects for GRADE outcomes by visual inspection of funnel plots when there were more than 10 trials. We planned to conduct sensitivity analyses for GRADE outcomes by examining only trials considered to have low risk of selection and detection bias. We conducted subgroup analyses to explore whether the effects of supplements differed with sex, SGA (birth weight <10th percentile), timing of supplementation (in hospital, after discharge, or both), primary feed (breastmilk or formula), or trial timing (conducted before or after 2000) and tested for interactions for GRADE outcomes (S1 Appendix). No unplanned analyses were performed.

## Results

**Fig 1:**
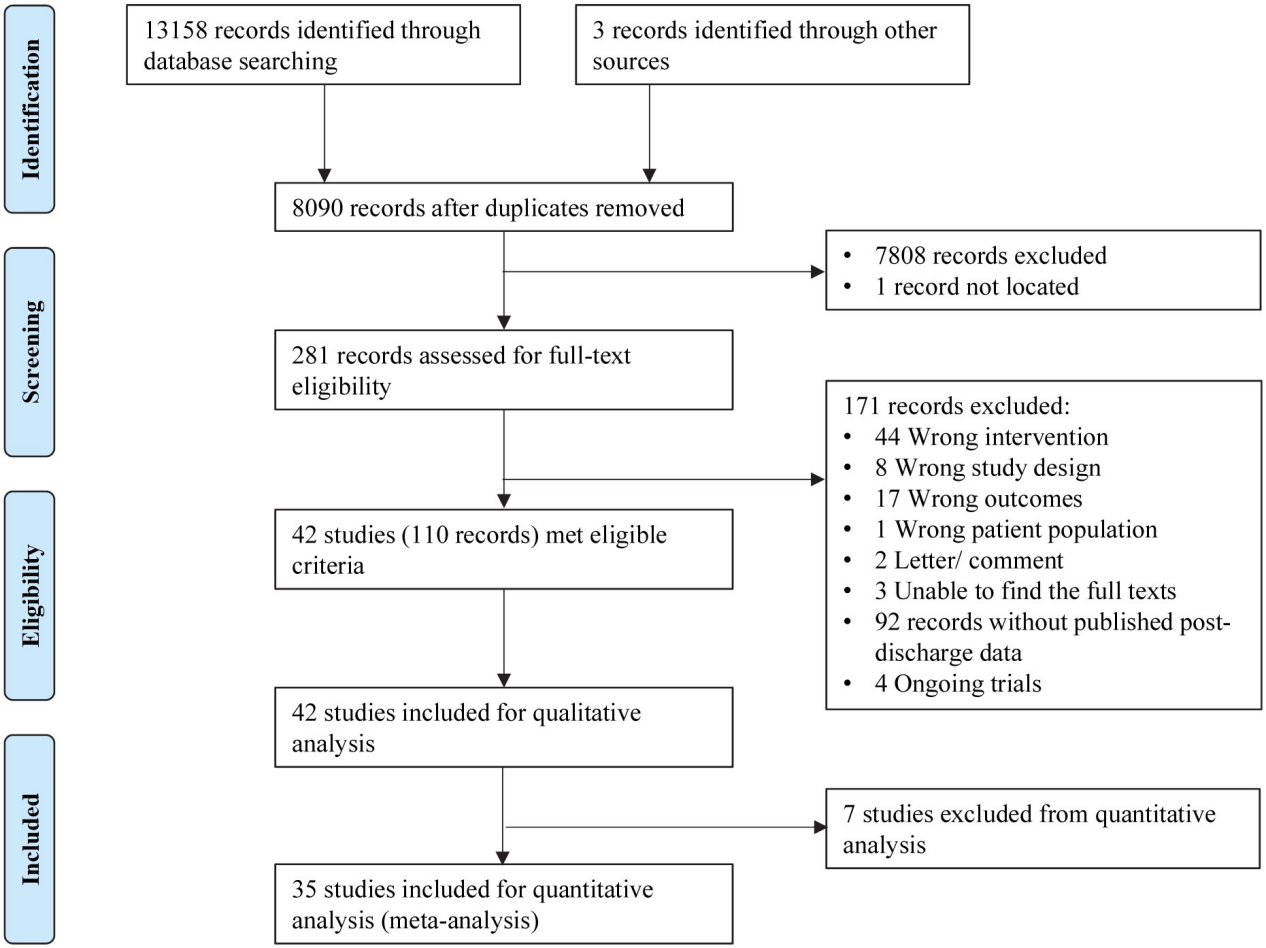
Flow diagram for included studies.

After deduplication, 8,090 records were identified. After title and abstract screening, we completed full-text screening for 281 records. We excluded 171 records that did not meet our inclusion criteria. We included the remaining 40 RCTs and 2 quasi-RCTs (118 records) in the qualitative analysis and 33 RCTs and 2 quasi-RCTs in the quantitative analysis (Fig 1, S2 Appendix). The included infants were born between 1972 and 2018. Three studies included term SGA infants [31–33], and the remaining studies included preterm infants. Infants in 13 studies received supplements in hospital [34–44], in 27 studies received supplements postdischarge [24,31,33,45–68], and in 2 studies received supplements both in hospital and postdischarge [32,69]. Twenty-two studies were conducted up to 2000 [24,31,32,35,37,38,41,43–45,47–52,56,60,61,66,69,70], and 20 were conducted in or after 2001 [33,34,36,39,40,42,46,53–55,57–59,62–65,67,68,71] (S2 Table).

### Risk of bias in included studies

Included studies were of variable methodological quality (S1a and S1b Fig), with around 45% having a high risk of attribution bias because of loss to long-term followup of more than 20%. Approximately 20% of included studies were at high risk of reporting bias because some prespecified outcomes were not reported [52,56,63,65,69] or the outcomes were only reported for some selected groups [37,44]. We considered that 17% of included studies were at high risk of performance bias because of lack of blinding of the families or clinicians. Although for many studies, blinding was not possible, e.g., when the intervention was breastfeeding [37,38,42–44], many did not comment on blinding of the outcome assessment. Nearly 15% of the included studies were at high risk of other bias because of imbalance between groups at baseline [31,32,34,57,58], different baseline data in each publication [24], or stopping early [39]. Two studies were at high risk of selection bias because the infants were quasirandomised according to their birth date [59] or last digit of the infant’s hospital number [70].

### Primary outcome: BMI in childhood

**Fig 2:**
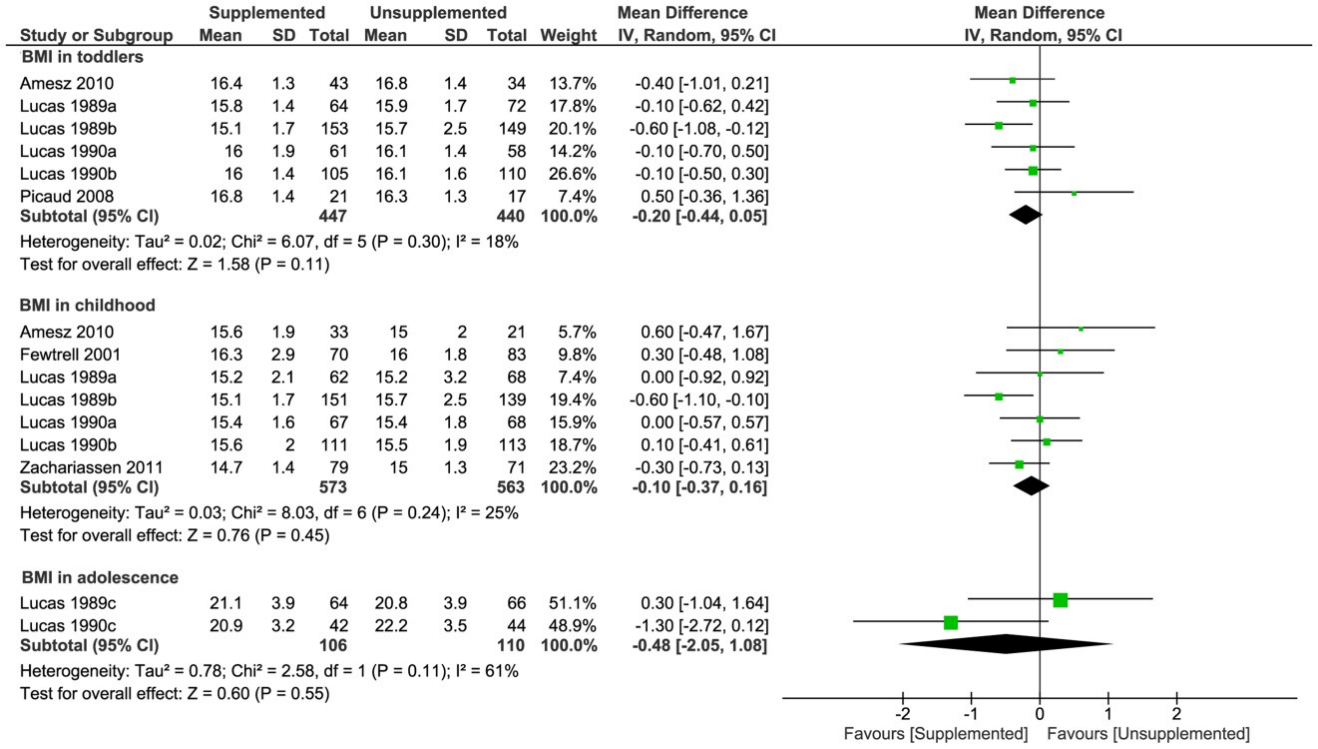
Forest plot of effect of macronutrient supplementation on BMI (kg/m^2^) in childhood (primary outcome), toddlers, and adolescence (secondary outcomes). *P*-values are from Z test for the summary effect and chi-squared test for heterogeneity. BMI, body mass index; CI; confidence interval.

In childhood, there was no significant difference in BMI between supplemented and unsupplemented groups (7 trials [32,37,44,46,67]; 1,136 children; MD [kg/m^2^] −0.10; 95% CI −0.37, 0.16; *P* = 0.45; Fig 2).

### Secondary outcomes

#### BMI

There were no significant differences between supplemented and unsupplemented groups in BMI in toddlers (6 trials [37,44,46,64]; 887 toddlers; MD [kg/m^2^] −0.20; 95% CI −0.44, 0.05; *P* = 0.11; Fig 2) or in adolescence (2 trials [37,44]; 216 adolescents; MD [kg/m^2^] −0.48; 95% CI −2.05, 1.08; *P* = 0.55; Fig 2).

#### Weight

**Fig 3:**
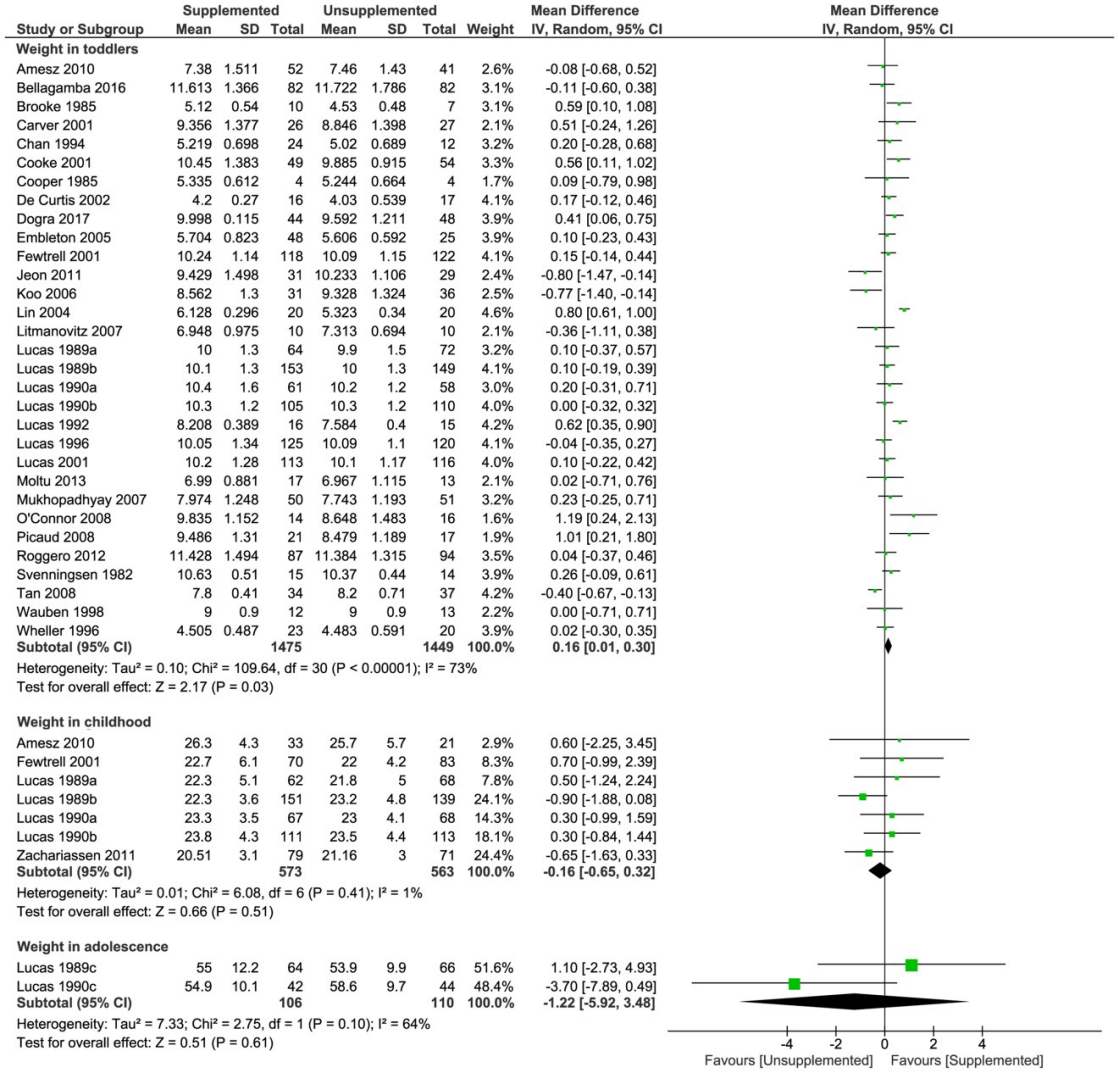
Forest plot of effect of macronutrient supplementation on weight (kg) in toddlers, childhood, and adolescence. *P*-values are from Z test for the summary effect and chi-squared test for heterogeneity. CI, confidence interval.

Supplementation increased weight in toddlers (31 trials [24,31–33,36–40,42–44,46,50–53,55,57–60,62,64–66,69,71]; 2,924 toddlers; MD [kg] 0.16; 95% CI 0.01, 0.30; *P* = 0.03; Fig 3). There were no significant differences between groups in weight in childhood (7 trials [32,37,44,46,67]; 1,136 children; MD [kg] −0.16; 95% CI −0.65, 0.32; *P* = 0.51; Fig 3) or in adolescence (2 trials [37,44]; 216 adolescents; MD [kg] −1.22; 95% CI −5.92, 3.48; *P* = 0.61; Fig 3). Sensitivity analysis including only studies at low risk of bias showed there was no significant difference in weight between toddlers in the supplemented and unsupplemented groups (MD [kg] 0.06; 95% CI −0.15, 0.27; *P* = 0.57; S2a Fig), and funnel plots (S3a Fig) did not suggest significant bias due to small study effects.

There were no significant differences between supplemented and unsupplemented groups in weight z-scores in toddlers (13 trials [34,39,40,42,46,47,49,55,57,58,61,67,68]; 1,135 toddlers; MD 0.01; 95% CI −0.27, 0.29; *P* = 0.94; S4a Fig), childhood (1 trial; 153 children; MD 0.14; 95% CI −0.23, 0.51; *P* = 0.46; S4a Fig), adolescence (2 trials [37,44]; 244 adolescents; MD −0.25; 95% CI −0.55, 0.05; *P* = 0.10; S4a Fig), or adulthood (2 trials [37,44]; 199 adults; MD −0.11; 95% CI −0.72, 0.50; *P* = 0.73; S4a Fig).

#### Length or height

**Fig 4:**
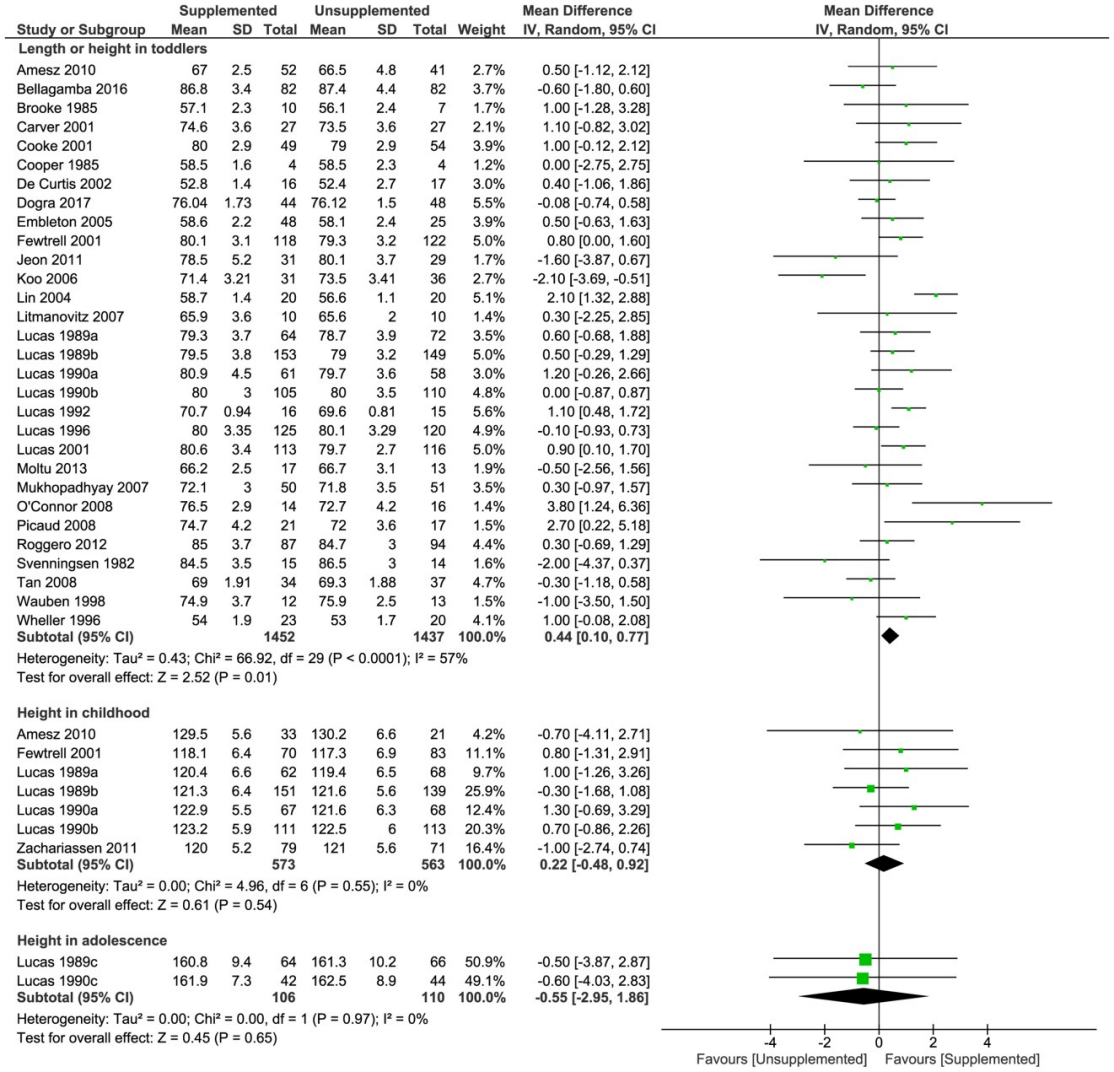
Forest plot of effect of macronutrient supplementation on length or height (cm) in toddlers, childhood, and adolescence. *P*-values are from Z test for the summary effect and chi-squared test for heterogeneity. CI, confidence interval.

Supplementation increased length in toddlers (30 trials [24,31–33,36–40,42–44,46,50,52,53,55,57–60,62,64–66,69,71]; 2,889 toddlers; MD [cm] 0.44; 95% CI 0.10, 0.77; *P* = 0.01; Fig 4). There were no significant differences between supplemented and unsupplemented groups in height in childhood (7 trials [32,37,44,46,67]; 1,136 children; MD [cm] 0.22; 95% CI −0.48, 0.92; *P* = 0.54; Fig 4) or in adolescence (2 trials [37,44]; 216 adolescents; MD [cm] −0.55; 95% CI −2.95, 1.86; *P* = 0.65; Fig 4). Sensitivity analysis including only studies at low risk of bias showed there was no significant difference between groups in length or height in toddlers (7 trials [32,36,37,42,44]; 1,175 toddlers; MD [cm] 0.28; 95% CI −0.08, 0.63; *P* = 0.13; S2b Fig) and funnel plots (S3b Fig) did not suggest significant bias due to small study effects.

There were no significant differences between supplemented and unsupplemented groups in length or height z-scores in toddlers (12 trials [34,39,40,42,46,49,55,57,58,61,67,68]; 1,083 toddlers; MD 0.08; 95% CI −0.19, 0.34; *P* = 0.58; S4b Fig), childhood (1 trial [32]; 153 children; MD 0.20; 95% CI −0.15, 0.55; *P* = 0.26; S4b Fig), or adulthood (2 trials [37,44]; 199 adults; MD −0.07; 95% CI −0.36, 0.22; *P* = 0.62; S4b Fig) but reduced height z-scores in adolescence (2 trials [35,70]; 244 adolescents; MD −0.30; 95% CI −0.57, −0.03; *P* = 0.03; S4b Fig).

#### Head circumference

**Fig 5:**
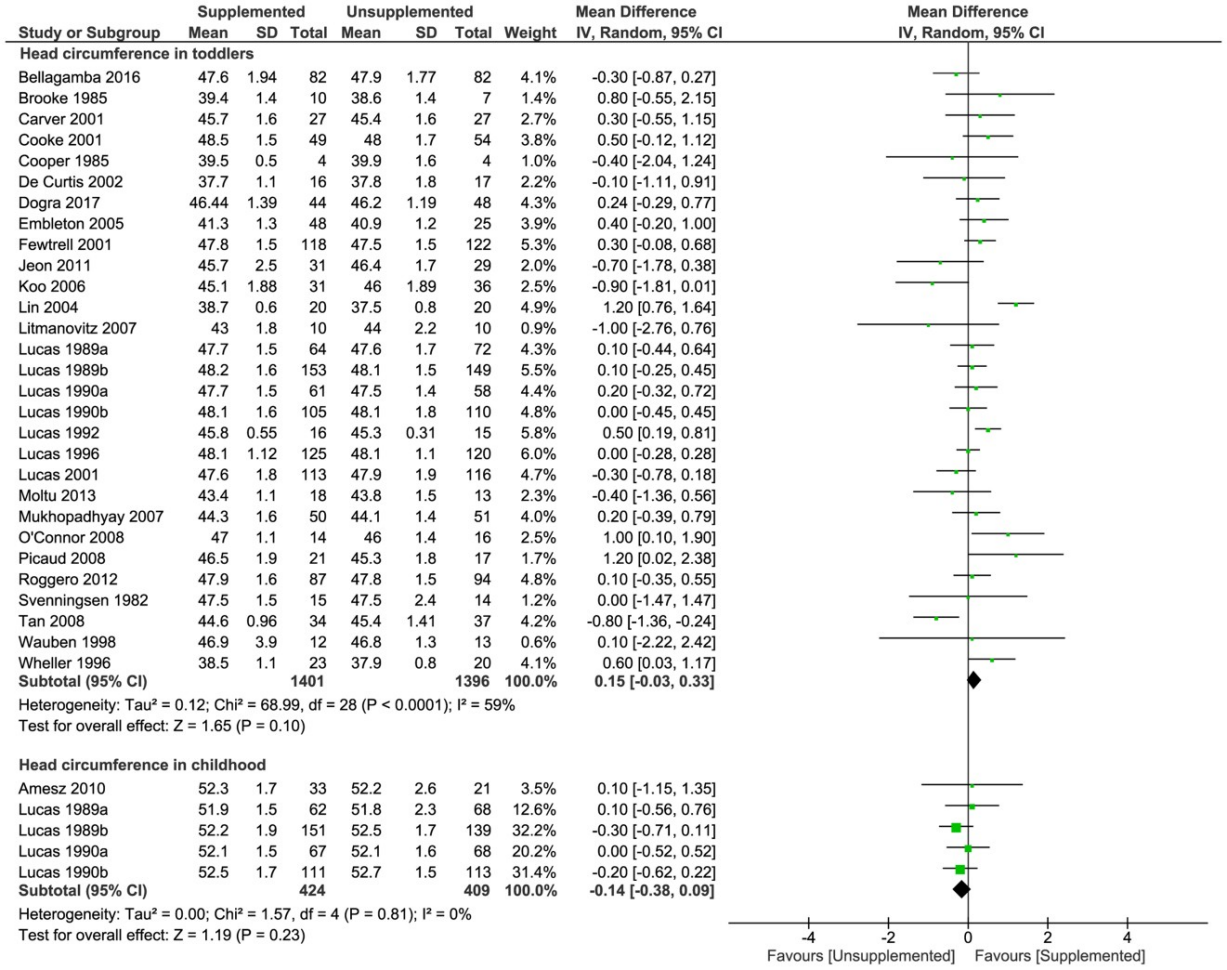
Forest plot of effect of macronutrient supplementation on head circumference (cm) in toddlers and childhood. *P*-values are from Z test for the summary effect and chi-squared test for heterogeneity. CI, confidence interval.

In toddlers, there were no significant differences between supplemented and unsupplemented groups in head circumference (29 trials [24,31–33,36–40,42–44,50,52,53,55,57–60,62,64–66,69,71]; 2,797 toddlers; MD [cm] 0.15; 95% CI −0.03, 0.33; *P* = 0.10; Fig 5). In childhood, there was no significant difference between groups in head circumference (5 trials [37,44,46]; 833 children; MD [cm] −0.14; 95% CI −0.38, 0.09; *P* = 0.23; Fig 5).

Sensitivity analysis including only studies at low risk of bias showed there was no evidence of differences in head circumference in toddlers between supplemented and unsupplemented nutrition groups (MD [cm] 0.04; 95% CI −0.20, 0.29; *P* = 0.72; S2c Fig), and funnel plots (S3c Fig) did not suggest significant bias due to small study effects.

Toddlers in the supplemented group had lower head circumference z-scores than toddlers in the unsupplemented group (10 trials [34,39,40,42,46,57,58,61,67,68]; 958 toddlers; MD −0.28; 95% CI −0.49 to −0.07; *P* = 0.008; S4c Fig).

#### Fat mass and lean mass

**Fig 6:**
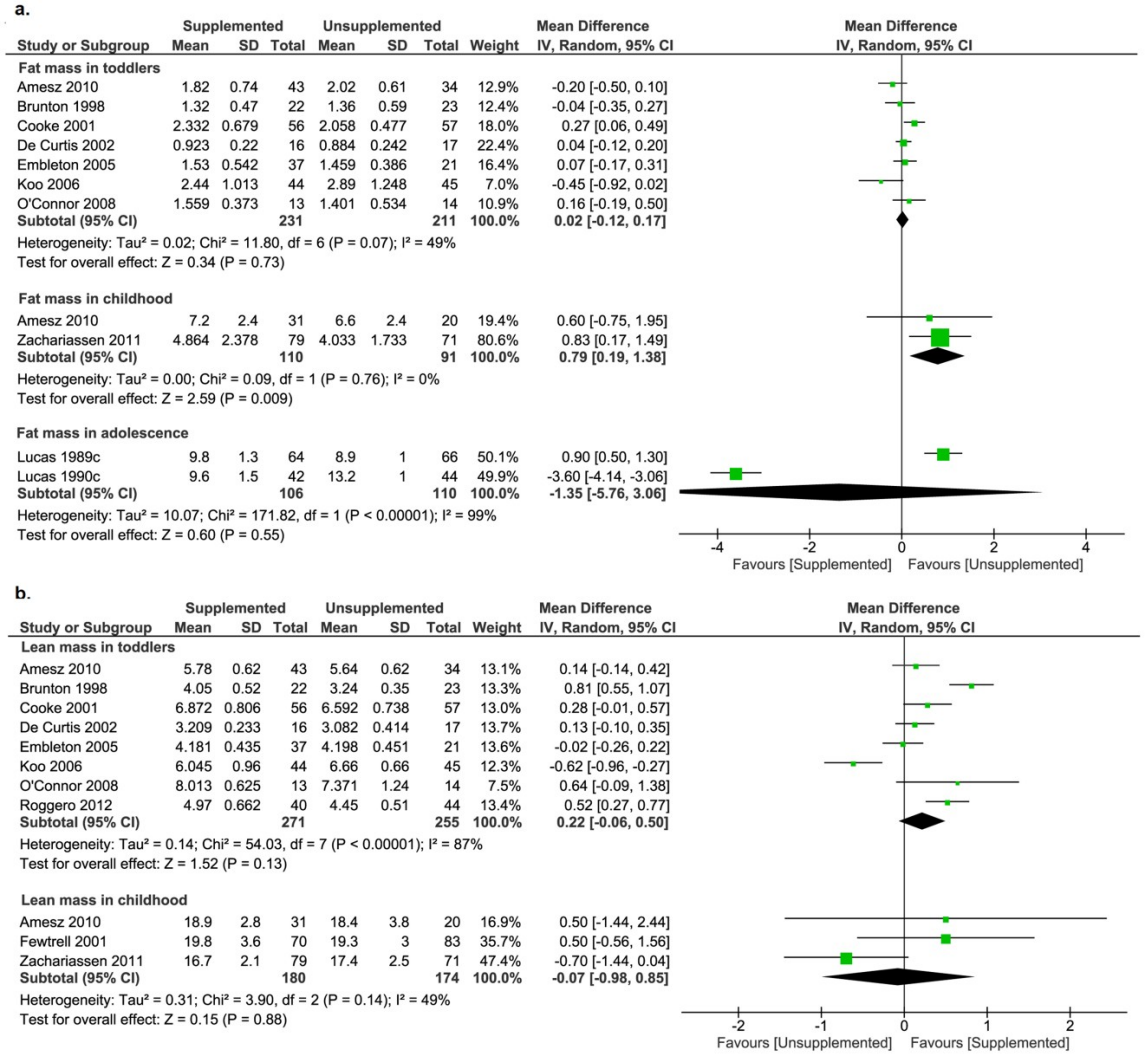
Forest plot of effect of macronutrient supplementation on fat mass and lean mass. *P*-values are from Z test for the summary effect and chi-squared test for heterogeneity. (a) Fat mass (kg); (b) Lean mass (kg). CI, confidence interval.

There were no significant differences between supplemented and unsupplemented groups in fat mass in toddlers (7 trials [24,46,49,53,55,58,62]; 442 toddlers; MD [kg] 0.02; 95% CI −0.12 to 0.17; *P* = 0.73; Fig 6). Supplementation increased fat mass in childhood (2 trials [46,67]; 201 children; MD [kg] 0.79; 95% CI 0.19, 1.38; *P* = 0.009; Fig 6), but not in adolescence (2 trials [37,44]; 216 adolescents; MD [kg] −1.35; 95% CI −5.76, 3.06; *P* = 0.55; Fig 6) or at >3 years (4 trials [37,44,46,67]; 417 children; MD [kg] −0.32; 95% CI −2.50, 1.85; *P* = 0.77; Fig 6). There were also no significant differences between supplemented and unsupplemented groups in lean mass in toddlers (8 trials [24,46,49,53,55,58,62,65]; 526 toddlers; MD [kg] 0.22; 95% CI −0.06, 0.50; *P* = 0.13; Fig 6) or in childhood (3 trials [32,46,67]; 354 children; MD [kg] −0.07; 95% CI −0.98, 0.85; *P* = 0.88; Fig 6).

#### Skin fold thickness

There were no significant differences between supplemented and unsupplemented groups in triceps skin fold thickness in toddlers (6 trials [37,44,61,70]; 1,021 toddlers; MD [mm] 0.11; 95% CI −0.12, 0.35; *P* = 0.35; S5a Fig) or in childhood (4 trials [37,44]; 779 children; MD [mm] −0.09; 95% CI −0.57, 0.38; *P* = 0.70; S5a Fig). There were also no significant differences between supplemented and unsupplemented groups in subscapular skin fold thickness in toddlers (6 trials [37,44,61,70]; 1,021 toddlers; MD [mm] −0.05; 95% CI −0.21, 0.10; *P* = 0.50; S5b Fig) or in childhood (4 trials [37,44]; 779 children; MD [mm] −0.10; 95% CI −0.64, 0.44; *P* = 0.71; S5b Fig).

#### Bone development

In studies that measured bone mineral content (BMC), toddlers in the supplemented group had higher BMC than those in the unsupplemented group (13 trials [24,41,43,46,47,49,51,53,55,58,60,62,64]; 670 toddlers; SMD 0.43; 95% CI 0.03, 0.83; *P* = 0.03; S6a Fig). There were no significant differences between groups in BMC in childhood (1 trial [46]; 51 children; SMD 0.11; 95% CI −0.45, 0.68; *P* = 0.69; S6a Fig), in adolescence (2 trials [37,44]; 239 adolescents; SMD −0.21; 95% CI −0.46, 0.05; *P* = 0.12; S6a Fig), or in adulthood (2 trials [37,44]; 199 adults; SMD −0.06; 95% CI −0.34, 0.22; *P* = 0.69; S6a Fig).

In studies that measured bone mineral density (BMD), the pattern was similar. Toddlers in the supplemented group had higher BMD than toddlers in the unsupplemented group (5 trials [24,41,55,62,64]; 289 children; SMD 0.40; 95% CI 0.07, 0.73; *P* = 0.02; S6b Fig). There were no significant differences between supplemented and unsupplemented groups in BMD in childhood (1 trial [46]; 51 children; SMD 0.00; 95% CI −0.56, 0.56; *P* = 1.00; S6b Fig), in adolescence (2 trials [37,44]; 239 adolescents; SMD −0.10; 95% CI −0.36, 0.15; *P* = 0.42; S6b Fig), or in adulthood (2 trials [37,44]; 199 adults; SMD −0.08; 95% CI −0.36, 0.21; *P* = 0.60; S6b Fig).

There were no data for the other secondary outcomes.

### Subgroup analyses

#### Sex of infants

There was no significant sex interaction for the effect of supplementation on BMI in childhood [67]. However, in toddlers, supplemented boys had greater length or height than unsupplemented boys (2 trials, 173 boys; MD [cm] 1.66, 95% CI 0.75, 2.58; *P* = 0.0003) [24,61], but there were no differences in girls (2 trials, 159 girls; MD [cm] 0.15, 95% CI −0.71, 1.01; *P* = 0.74) (*P* = 0.02 for interaction). There were no significant sex interactions for weight or head circumference in toddlers (Table 1).

**Table 1:** Summary of subgroup analyses.

	Subgroup	Number of Participants (Studies)	MD [95% CI]	*P* for Overall Effect	I^2^	*P* for Subgroup Interaction
**Sex of infants**						
BMI in childhood	boys	70 (1 RCT) [67]	−0.60 [−1.13, −0.07] kg/m^2^	0.03	NA	0.16
	girls	80 (1 RCT) [67]	0.00 [−0.66, 0.66] kg/m^2^	1.00	NA	
Weight in toddlers	boys	173 (2 RCTs) [24,61]	0.59 [−0.13, 1.32] kg	0.11	73%	0.10
	girls	159 (2 RCTs) [24,61]	−0.08 [−0.43, 0.26] kg	0.63	0%	
Length/height in toddlers	boys	173 (2 RCTs) [24,61]	1.66 [0.75, 2.58] cm	0.0003	0%	0.02
	girls	159 (2 RCTs) [24,61]	0.15 [−0.71, 1.01] cm	0.74	0%	
Head circumference in toddlers	boys	173 (2 RCTs) [24,61]	0.16 [−1.41, 1.73] cm	0.84	87%	0.68
	girls	159 (2 RCTs) [24,61]	−0.18 [−0.65, 0.29] cm	0.45	0%	
**SGA infants**						
BMI in childhood	SGA infants	153 (1 RCT) [32]	0.30 [−0.48, 1.08] kg/m^2^	0.45	NA	NA
Weight in toddlers	SGA infants	370 (4 RCTs) [31–33,65]	0.40 [−0.04, 0.83] kg	0.07	83%	NA
Length/height in toddlers	SGA infants	370 (4 RCTs) [31–33,65]	0.97 [−0.10, 2.05] cm	0.08	73%	NA
Head circumference in toddlers	SGA infants	370 (4 RCTs) [31–33,65]	0.51 [−0.15, 1.17] cm	0.13	81%	NA
Height in childhood	SGA infants	153 (1 RCT) [32]	0.80 [−1.31, 2.91] cm	0.46	NA	NA
**Timing of supplements**						
BMI at >3 years (childhood)	started in the hospital	779 (4 RCTs) [37,44]	−0.16 [−0.52, 0.20] kg/m^2^	0.39	32%	0.57
	across in-hospital and postdischarge periods	153 (1 RCT) [32]	0.30 [−0.48, 1.08] kg/m^2^	0.45	NA	
	started after hospital discharge	204 (2 RCTs) [46,67]	−0.10 [−0.83, 0.85] kg/m^2^	0.99	25%	
Weight in toddlers	started in the hospital	1,573 (12 RCTs) [36–40,42–44,55,71]	0.03 [−0.1, 0.17] kg	0.65	34%	0.24
	across in-hospital and postdischarge periods	269 (2 RCTs) [32,69]	0.20 [−0.03, 0.42] kg	0.08	0%	
	started after hospital discharge	1,082 (17 RCTs) [24,31,33,46,50–53,57–62,64–66]	0.16 [0.01, 0.30] kg	0.03	73%	
Length or height in toddlers	started in the hospital	1,573 (12 RCTs) [36–40,42–44,55,71],	0.08 [−0.22, 0.37] cm	0.60	0%	0.07
	across in-hospital and postdischarge periods	269 (2 RCTs) [32,69]	−0.37 [−3.08, 2.34] cm	0.79	79%	
	started after hospital discharge	1,047 (16 RCTs) [24,31,33,46,50,52,53,57–62,64–66]	0.79 [0.25, 1.33] cm	0.004	60%	
Head circumference in toddlers	started in the hospital	1,573 (12 RCTs) [36–40,42–44,55,71],	0.01 [−0.16, 0.17] cm	0.93	19%	0.19
	across in-hospital and postdischarge periods	269 (2 RCTs) [32,69]	0.28 [−0.09, 0.65] cm	0.13	0%	
	started after hospital discharge	954 (15 RCTs) [24,31,33,50,52,53,57–62,64–66]	0.28 [−0.05, 0.60] cm	0.10	68%	
Fat mass at >3 years	started in the hospital	216 (2 RCTs) [37,44]	−1.34 [−5.75, 3.07] kg	0.55	99%	0.35
	started after hospital discharge	201 (2 RCTs) [46,67]	0.79 [0.19, 1.38] kg	0.009	0%	
Lean mass at >3 years (childhood)	across in-hospital and postdischarge periods	153 (1 RCT) [32]	0.5 [−0.56, 1.56] kg	0.36	NA	0.11
	started after hospital discharge	354 (2 RCTs) [46,67]	−0.55 [−1.24, 0.15] kg	0.12	22%	
Height at >3 years (childhood)	started in the hospital	779 (4 RCTs) [37,44]	0.47 [−0.38, 1.32] cm	0.28	0%	0.25
	across in-hospital and postdischarge periods	153 (1 RCT) [32]	0.80 [1.31, 2.91] cm	0.46	NA	
	started after hospital discharge	204 (2 RCTs) [46,67]	−0.94 [−2.48, 0.61] cm	0.23	0%	
**Primary feed**						
BMI at >3 years (childhood)	formula as primary milk feed	566 (4 RCTs) [32,44,46]	0.15 [−0.18, 0.48] kg/m^2^	0.37	0%	0.02
	breast milk as primary milk feed	570 (3 RCTs) [37,67]	−0.38 [−0.69, −0.07] kg/m^2^	0.01	0%	
Weight in toddlers	formula as primary milk feed	1,758 (22 RCTs) [24,31–33,44,46,50–53,55,57–62,64–66,69]	0.21 [0.04, 0.38] kg	0.02	73%	0.001
	breast milk as primary milk feed	901 (6 RCTs) [36–38,40,43]	0.14 [−0.02, 0.29] kg	0.09	0%	
	parenteral and enteral nutrition	265 (3 RCTs) [39,42,71]	−0.30 [−0.52, −0.08] kg	0.008	0%	
Length or height in toddlers	formula as primary milk feed	1,723 (22 RCTs) [24,31–33,44,46,50,52,53,55,57–62,64–66,69]	0.67 [0.24, 1.11] cm	0.003	58%	0.02
	breast milk as primary milk feed	901 (6 RCTs) [36–38,40,43]	0.13 [−0.26, 0.52] cm	0.51	0%	
	parenteral and enteral nutrition	265 (3 RCTs) [39,42,71]	−0.42 [−1.09, 0.26] cm	0.23	0%	
Head circumference in toddlers	formula as primary milk feed	1,630 (20 RCTs)[24,31–33,44,50,52,53,55,57–62,64–66,69]	0.27 [0.04, 0.50] cm	0.02	59%	0.001
	breast milk as primary milk feed	901 (6 RCTs) [36–38,40,43]	0.80 [−0.10, 0.26] cm	0.36	0%	
	parenteral and enteral nutrition	265 (3 RCTs) [39,42,71]	−0.53 [−0.90, -0.16] cm	0.005	0%	
Height at >3 years (Childhood)	formula as primary milk feed	566 (4 RCTs) [32,44,46]	0.75 [−0.26, 1.77] cm	0.14	0%	0.15
	breast milk as primary milk feed	570 (3 RCTs) [37,67]	−0.28 [−1.25, 0.70] cm	0.58	0%	
Fat mass at >3 years	formula as primary milk feed	137 (2 RCTs) [44,46]	−1.54 [−5.56, 2.58] kg	0.46	97%	0.25
	breast milk as primary milk feed	280 (2 RCTs) [37,67]	0.88 [0.49, 1.26] kg	<0.001	0%	
Lean mass at >3 years (Childhood)	formula as primary milk feed	204 (2 RCTs) [32,46]	0.5 [−0.43, 1.43] kg	0.29	0%	0.05
	breast milk as primary milk feed	150 (1 RCT) [67]	−0.70 [−1.44, 0.04] kg	0.06	NA	
**Different timing**						
BMI at >3 years (childhood)	before or in 2000	932 (5 RCTs) [32,37,44]	−0.09 [−0.42, 0.24] kg/m^2^	0.58	29%	0.82
	after 2000	204 (2 RCTs) [46,67]	0.01 [−0.83, 0.85] kg/m^2^	0.98	57%	
Weight in toddlers	before or in 2000	1,831 (16 RCTs) [24,31,32,37,38,43,44,50–52,60,61,66,69]	0.20 [0.09, 0.32] kg	0.0006	29%	0.45
	after 2000	1,093 (15 RCTs) [33,36,39,40,42,46,53,55,57–59,62,64,65,71]	0.09 [−0.19, 0.36] kg	0.54	84%	
Length or height in toddlers	before or in 2000	1,796 (15 RCTs) [24,31,32,37,38,43,44,50,52,60,61,66,69]	0.62 [0.32, 0.92] cm	<0.001	14%	0.37
	after 2000	1,093 (15 RCTs) [33,36,39,40,42,46,53,55,57–59,62,64,65,71]	0.30 [−0.34, 0.93] cm	0.36	71%	
Head circumference in toddlers	before or in 2000	1,796 (15 RCTs) [24,31,32,37,38,43,44,50,52,60,61,66,69]	0.19 [0.05, 0.32] cm	0.007	9%	0.55
	after 2000	1,001 (14 RCTs) [33,36,39,40,42,46,53,55,57–59,62,64,65,71]	0.06 [−0.32, 0.44] cm	0.75	76%	
Height at >3 years (Childhood)	before or in 2000	932 (5 RCTs) [32,37,44]	0.52 [−0.27, 1.31] cm	0.20	0%	0.10
	after 2000	204 (2 RCTs) [46,67]	−0.94 [−2.48, 0.61] cm	0.23	0%	
Fat mass at >3 years	before or in 2000	280 (2 RCTs) [37,44]	0.88 [0.49, 1.26] kg	<0.001	0%	0.25
	after 2000	137 (2 RCTs) [46,67]	−1.54 [−5.56, 2.58] kg	0.46	97%	
Lean mass at >3 years (Childhood)	before or in 2000	153 (1 RCT) [32]	0.50 [−0.56, 1.56] kg	0.36	NA	0.20
	after 2000	201 (2 RCTs) [46,67]	−0.45 [−1.41, 0.51] kg	0.36	22%	

**Abbreviations**: BMI, body mass index; CI, confidence interval; MD, mean difference; NA, not applicable; RCT, randomised controlled trial; SGA, small for gestational age.

*P*-values are from Z test for the summary effect and chi-squared test for heterogeneity.

#### SGA infants

In children born SGA, there were no significant differences between supplemented and unsupplemented groups in BMI in childhood [32], weight in toddlers [31–33,65], length or height in toddlers [31–33,65], head circumference in toddlers [31–33,65], or height in childhood [32] (Table 1).

#### Timing of supplements

There were no significant differences between supplemented and unsupplemented groups in the different timing subgroups for BMI in childhood, head circumference in toddlers, lean mass in childhood, or height in childhood and no evidence of an interaction between timing and effects of supplements. Toddlers who had received supplements after hospital discharge, but not if they received supplements both in hospital and postdischarge or only in hospital, were heavier and longer than those who had not received supplements (weight: 17 trials [24,31,33,46,50–53,57–62,64–66], 1,082 toddlers; MD [kg] 0.16, 95% CI 0.01, 1.30, *P* = 0.03; length or height: 16 trials [24,31,33,46,50,52,53,57–62,64–66], 1,047 toddlers; MD [cm] 0.79, 95% CI 0.25, 1.33; *P* = 0.004) and had greater fat mass in childhood (2 trials [46,67], 201 children; MD [kg] 0.79, 95% CI 0.19, 1.38; *P* = 0.009), but there was no evidence of an interaction between timing of supplements and effects on these outcomes (Table 1).

#### Primary feed

In children who had received formula as their primary feed, there were no significant differences between the supplemented and the unsupplemented groups in BMI in childhood [32,44,46], height at >3 years [32,44,46], fat mass at >3 years [44,46], and lean mass at >3 years [32,46] (Table 1). However, supplemented toddlers had greater weight (22 trials [24,31–33,44,46,50–53,55,57–62,64–66,69], 1,758 toddlers; MD [kg] 0.21; 95% CI 0.04, 0.38; *P* = 0.02), length or height (22 trials [24,31–33,44,46,50,52,53,55,57–62,64–66,69], 1,723 toddlers; MD [cm] 0.67; 95% CI 0.24, 1.11; *P* = 0.003), and head circumference (20 trials [24,31–33,44,50,52,53,55,57–62,64–66,69], 1,630 toddlers; MD [cm] 0.27, 95% CI 0.04, 0.50; *P* = 0.02).

In children who had received breast milk as their primary feed, children in the supplemented groups had lower BMI in childhood (3 trials [37,67], 570 children; MD [kg/m^2^] −0.38; 95% CI −0.69, −0.07; *P* = 0.01) but higher fat mass at >3 years (2 trials [37,67], 280 children; MD [kg] 0.88; 95% CI 0.49, 1.26; *P* < 0.001), and there were no significant differences between groups in lean mass [67] or height [37,67] at >3 years (Table 1). In toddlers, there were no significant differences between groups in weight [36–38,40,43], length or height [36–38,40,43], and head circumference [36–38,40,43].

In toddlers who had received parenteral and enteral feed as primary feed, the supplemented groups had lower weight (3 trials [39,42,71], 265 toddlers; MD [kg] −0.30; 95% CI −0.52, −0.08; *P* = 0.008) and head circumference (3 trials [39,42,71], 265 toddlers; MD [cm] −0.53; 95% CI −0.90, −0.16; *P* = 0.005), but not length or height [39,42,71], than the unsupplemented groups (Table 1).

The tests for an interaction between primary feed and macronutrient supplements were significant for BMI in childhood (*P* = 0.02), weight (*P* = 0.001), length or height (*P* = 0.02), and head circumference (*P* = 0.001) in toddlers (Table 1). There was no evidence of an interaction between primary feed and effects of supplements on height, fat mass, or lean mass at >3 years.

#### Trial timing

In the different trial timing subgroups, there were no significant differences between supplemented and unsupplemented groups in BMI in childhood. In trials conducted before or in 2000, toddlers in the supplemented group had greater weight (16 trials [24,31,32,37,38,43,44,50–52,60,61,66,69], 1,831 toddlers; MD [kg] 0.20; 95% CI 0.09, 0.32; *P* = 0.0006), length or height (15 trials [24,31,32,37,38,43,44,50,52,60,61,66,69], 1,796 toddlers; MD [cm] 0.62; 95% CI 0.32, 0.92; *P* < 0.001), and head circumference (15 trials [24,31,32,37,38,43,44,50,52,60,61,66,69], 1,796 toddlers; MD [cm] 0.19; 95% CI 0.05, 0.32; *P* = 0.007) than toddlers in the unsupplemented group. However, there were no significant differences between supplemented and unsupplemented groups in weight [33,36,39,40,42,46,53,55,57–59,62,64,65,71], length or height [33,36,39,40,42,46,53,55,57–59,62,64,65,71], and head circumference [33,36,39,40,42,46,53,55,57–59,62,64,65,71] in toddlers in the trials conducted after 2000 (Table 1). There was no evidence of an interaction between trial timing and macronutrient supplements on BMI in childhood, weight in toddlers, length in toddlers, head circumference in toddlers, or height at >3 years.

### Studies not included in quantitative synthesis

Agosti and colleagues [45] compared infants who received preterm formula (*n* = 69) or standard term formula (*n* = 52) after hospital discharge. At 12 months’ corrected age, they reported no significant differences in weight (mean 9.7 kg versus 9.3 kg), length (mean 75.6 cm versus 74.7 cm), head circumference (mean 46.4 cm versus 46.1cm), and BMI between groups. In the subgroup of SGA infants, supplemented infants had greater length (mean 74.6 cm versus 71.6 cm) than unsupplemented infants at 12 months’ corrected age. Data were presented in figures, and no standard deviations were reported.

Bhatia and colleagues [48] compared infants who received standard infant formula or reduced protein formula (total *n* = 24) after hospital discharge. There were no differences in growth parameters (weight, length, and head circumference) between groups. Data were not presented, and the numbers of infants in each group were not reported.

Cooper and colleagues [70] compared infants who received preterm formula (*n* = 10) or standard formula (*n* = 10). There were no significant differences in weight, length, head circumference, and skin fold thickness between groups. Data were presented as percent of the expected values.

Davies [35] compared growth rate (weight, length, head circumference) between infants who received supplemented formula (*n* = 34) or breast milk (*n* = 34). There were no differences in growth rates between groups from 1 to 2 months after birth. The absolute growth data were presented in figures, and no standard deviations were reported.

Ekcharoen and colleagues [54] compared growth rate (weight, length, head circumference) between infants who received postdischarge formula (*n* = 6) or high-protein, medium-chain triglyceride formula (*n* = 5). There were no differences in growth rates between groups, but measurements were not presented.

Friel and colleagues [56] compared infants who received enriched formula (*n* = 27) or standard term formula (*n* = 27). There were no significant differences in growth rate between groups, but measurements were not presented.

Peng and colleagues [63] compared infants who received preterm formula (*n* = 19) or standard term formula (*n* = 15). There were no significant differences in weight, length, or head circumference between groups at 6 months’ corrected age. Data were presented in figures, and no standard deviations were reported.

### Quality of evidence (GRADE)

The quality of the evidence was assessed as low or very low for all the GRADE outcomes (Table 2).

**Table 2:** GRADE table: Summary of findings.

**Supplemented compared to unsupplemented nutrition for children born preterm or SGA**				
**Patient or population**: children born preterm or SGA				
**Setting**: hospital or NICU				
**Intervention**: supplemented nutrition				
**Comparison**: unsupplemented nutrition				
**Outcomes**	Anticipated Absolute Effects* **(95% CI)**			**Grade of evidence**
	Risk with Unsupplemented Nutrition	Risk with Supplemented Nutrition		
BMI at >3 years (childhood)	Comparator	The mean BMI at >3 years (childhood) in the intervention group was 0.1 kg/m^2^ lower (0.37 lower to 0.16 higher)	1,136 (7 RCTs)	⨁⨁◯◯LOW^c^^,^^d^
Weight in toddlers (kg)	Comparator	The mean weight in toddlers in the intervention group was 0.16 kg higher (0.01 higher to 0.30 kg higher)	2,924 (31 RCTs)	⨁◯◯◯VERY LOW^a^^,^^b^^,^^c^
Head circumference in toddlers (cm)	Comparator	The mean head circumference in toddlers in the intervention group was 0.15 cm higher (0.03 lower to 0.33 cm higher)	2,797 (29 RCTs)	⨁◯◯◯VERY LOW^a^^,^^b^^,^^c^
Length/height in toddlers (cm)	Comparator	The mean length/height in toddlers in the intervention group was 0.44 cm higher (0.1 higher to 0.77 cm higher)	2,889 (30 RCTs)	⨁◯◯◯VERY LOW^a^^,^^b^^,^^c^
Height at >3 years (cm) (childhood)	Comparator	The mean height at >3 years (childhood) in the intervention group was 0.22cm higher (0.48 lower to 0.92 cm higher)	1,136 (7 RCTs)	⨁⨁◯◯LOW^c^^,^^d^
Fat mass at >3 years (kg)	Comparator	The mean fat mass at >3 years in the intervention group was 0.33 kg lower (2.78 lower to 2.12 kg higher)	417 (4 RCTs)	⨁◯◯◯VERY LOW^b^^,^^c^^,^^d^
Lean mass at >3 years (kg) (childhood)	Comparator	The mean lean mass at >3 years (childhood) in the intervention group was 0.07 kg lower (0.98 lower to 0.85 kg higher)	354 (3 RCTs)	⨁⨁◯◯LOW^c^^,^^d^

*The risk in the intervention group (and its 95% CI) is based on the assumed risk in the comparison group and the relative effect of the intervention (and its 95% CI).

**Abbreviations**: BMI, body mass index; CI, confidence interval; GRADE, Grading of Recommendations Assessment, Development and Evaluation; MD, mean difference; NICU, Newborn Intensive Care Unit; RCT, randomised controlled trial; RR, risk ratio; SGA, small for gestational age.

GRADE Working Group grades of evidence: high certainty, we are very confident that the true effect lies close to that of the estimate of the effect; moderate certainty, we are moderately confident in the effect estimate—the true effect is likely to be close to the estimate of the effect, but there is a possibility that it is substantially different; low certainty, our confidence in the effect estimate is limited—the true effect may be substantially different from the estimate of the effect; very low certainty, we have very little confidence in the effect estimate—the true effect is likely to be substantially different from the estimate of effect.

^a^Studies were supported by formula or fortifier companies whose role was not specified.

^b^Large losses to followup at 6 to 8 years.

^c^Uncertainty about methods used to generate a random sequence, conceal allocation, or blind outcome assessors.

^d^Substantial heterogeneity existed.

## Discussion

In our systematic review and meta-analysis of 40 RCTs and 2 quasi-RCTs involving 4,352 infants born preterm or SGA, we found no evidence that early macronutrient supplements led to significant differences in BMI in childhood. However, consistent with our hypothesis, we found that early macronutrient supplements may increase weight and length in toddlers, although the effect is inconsistent and unlikely to be clinically significant, and also increase bone mineralisation. Macronutrient supplements did not increase head circumference, and none of these effects persisted into later life. We also found that early supplements may increase fat mass in childhood, but not in adolescence.

Previous literature has suggested the possible early nutritional origins of obesity. An observational study indicated that faster weight gain in early life is associated with higher fat mass in later life [72]. Another retrospective study also demonstrated that higher average protein intake during initial hospitalisation was associated with increased fat mass at 9.5 years of age in children born preterm [73]. We chose BMI as the primary outcome for this analysis, on the assumption that this would be the most widely available measure of adiposity and is predictive of later cardiometabolic risk. Our data show that early supplements had no effect on BMI or lean mass in childhood and BMI in adolescence and no effect on skin fold thickness in toddlers or childhood. Although supplements may increase fat mass in childhood, limited data suggest that this does not persist in adolescence or in the combined group of >3 years. Therefore, our findings from randomised trials challenge the previous observational reports that early supplements lead to faster early growth and also increased later adiposity.

In toddlers, the supplemented group had greater weight and length or height but similar head circumference when compared to those in the unsupplemented nutrition group. However, in the sensitivity analyses including only trials at low risk of bias, there were no differences between groups in weight, length or height, and head circumference in toddlers. In the subgroup of trials reporting z-scores, toddlers in the supplemented nutrition group also had similar weight and height/length z-scores but reduced head circumference z-scores. Several factors may contribute to these apparent discrepancies between findings using absolute values and those using z-scores. Firstly, the analyses of absolute values and z-scores included different trials. For instance, only 8 trials reported both absolute values and z-scores for weight in toddlers, and the differences between groups were in the same direction for both outcomes in all of these trials. Four trials only reported weight z-scores, and 22 trials only reported absolute values.

Secondly, in 4 of 12 trials reporting z-scores, infants in the unsupplemented groups were larger than those in the supplemented nutrition groups at followup. In 3 of these trials [34,57,58], infants in the unsupplemented group had greater gestational age at study entry, and in 1 trial [42], infants in the unsupplemented group who were followed up were of greater gestational age and birth weight because of loss of followup. Excluding these 4 trials with substantially dissimilar group characteristics (24% of the total children reporting z-scores), the findings for z-scores were similar to those for absolute measures (i.e., supplemented groups had higher weight z-score and length z-scores, and similar head circumference z-scores, compared with the unsupplemented groups).

Further, heterogeneity was high for both sets of analyses. The wide range of ages (from 3 months to 24 months) may contribute to heterogeneity in the analyses of absolute growth values, whereas z-scores are age- and sex-specific, which would be expected to result in narrower CIs. However, only a few studies reported z-scores. We would recommend reporting both absolute and z-score values in future studies.

Moreover, a much larger number of trials and participants have been included in the quantitative analysis of absolute growth measurements than those of z-scores. Statistical significance is heavily dependent on the sample size, so when the sample size is large, even small treatment effects can appear statistically significant [74]. We conclude that, given the lack of effect of supplementation on growth measurements in the subgroup of low-risk studies or in those reporting z-scores, the small apparent effect of macronutrient supplementation on absolute weight (MD 0.16 kg) and length or height (0.44 cm) in toddlers is unlikely to be clinically significant.

Our data also suggest that toddlers in the supplemented group had greater bone mineralisation than infants in the unsupplemented group. Approximately 80% of total body bone mineralisation of the fetus is acquired in the last trimester of pregnancy [10–12], so preterm infants are at risk for suboptimal bone mineralisation. Deficits in mineralisation during this period could increase risk of childhood fracture and cause reduced peak bone mass [75,76], which may result in higher risk of osteoporosis in later life [77]. Thus, providing adequate nutritional support to achieve adequate bone accretion in early life may be of initial clinical benefit for infants born small. However, limited evidence from this systematic review suggests that early macronutrient supplementation does not affect long-term bone mineralisation.

In order to further explore sources of heterogeneity in key outcomes, we undertook a number of prespecified subgroup analyses. We specifically hypothesised that the effects of supplements may be different in girls and boys. We found that supplementation increased length or height in toddler boys, but not girls, suggesting that the effects of nutrient supplements may be sex-specific, although there were no data to show whether these differences persisted in later life. Animal studies have shown some evidence of sex differences in these effects, with preterm male lambs who received early supplemented nutrition having greater early weight gain and increased ponderal index in adulthood, effects that were not seen in females [78]. Male, but not female, preterm piglets receiving a high-protein diet also had higher growth rates than those receiving an adequate protein diet after weaning [79].

We anticipated that the effect of supplementation on growth may also depend in part upon the primary feed being supplemented (formula versus breastmilk versus parenteral nutrition). Intriguingly, supplements appeared to increase early growth in weight, length, and head circumference only if the primary feed was formula, but not if the primary feed was breastmilk, whereas supplements provided as both parenteral and enteral feeds decreased toddler weight and head circumference. A previous systematic review also reported that feeding preterm infants with formula is associated with faster in-hospital rates of growth [80]. Our review shows that the effect of nutrient-enriched formula on growth may persist after hospital discharge but not last through childhood and adolescence.

However, the reason for the lack of effect of supplements on early growth if breastmilk was the primary feed is not clear and raises the interesting possibility that the previously reported protective effect of breastmilk for later obesity [81,82] may also apply to early growth acceleration. However, this conflicts with our findings that supplemented breast milk decreased BMI but increased fat mass in childhood. Another possible explanation may be that infants whose primary feed was breastmilk, which is generally of lower calorie and protein content than formula, received less total nutrition or less additional nutrition in the supplemented group. However, our estimates showed that infants in the supplemented group who received breastmilk as their primary feed had similar energy intakes as those whose primary feed was formula, making this explanation unlikely (S3 Table).

In subgroup analysis, it appeared that providing both parenteral and enteral supplements had an adverse effect on early growth. However, in one trial, infants in the unsupplemented group who were followed up were of greater gestational age and birthweight than those in the supplemented group, and after exclusion of this trial, there was no effect of parenteral and enteral supplements on early growth. Therefore, we are unable to draw any conclusions about the effect of combination parenteral and enteral feed.

We found that toddler weight, length, and head circumference increased in supplemented infants if they received postdischarge nutrition, but these effects were not seen in the other 2 timing groups, although this interaction term was not significant. It is possible that introducing supplements before discharge has little effect on later size because illness and factors other than nutrient intake limit growth at this time, as opposed to after discharge, when infants are in the phase of catch-up growth. It is also possible that the effects of supplementation were only transient in both groups but that growth outcomes after discharge were measured some time after the supplementation for infants who received in-hospital supplements but much closer to the time of supplementation for infants who received postdischarge supplements.

In order to explore other sources of heterogeneity, we carried out subgroup analyses according to the study date. We found that supplements increased growth in toddlers in studies conducted before and during 2000, but not in studies conducted after 2000, although the interaction term was not significant. Possible reasons for these differences may be the wide variations in estimating the values of macronutrient composition of preterm human milk and different neonatal intensive care units and commercial companies using different values to modify the composition of formula and fortifier over time [83,84]. We expected that differences in baseline (unsupplemented) intakes over time may contribute to differences in the overall effects of macronutrient supplements, with later studies potentially reporting higher macronutrient intakes in the unsupplemented groups and hence smaller effects of additional supplements compared to earlier studies. However, our analyses indicated that there were no differences in mean baseline intakes or in MDs between supplemented and unsupplemented nutrition groups between the 2 epochs (S4 Table).

In the subgroup of infants born SGA, there were no differences in BMI and height in childhood or in weight, length, and head circumference as toddlers between supplemented and unsupplemented groups, but there was substantial heterogeneity in these analyses. Of the trials reporting growth in toddlers born SGA, 3 studied term SGA infants [31–33] and reported greater weight, length, and head circumference in the supplemented groups, whereas 1 trial studied preterm SGA infants [65] and reported no difference in these outcomes between supplemented and unsupplemented groups. Term and preterm SGA infants may respond differently to early supplements, and further studies are needed to explore the interactions between gestational age and macronutrient supplements in SGA infants.

Previously published systematic reviews [17,25,26] of the effects of macronutrient supplements for preterm infants only reported growth outcomes up to 18 months of age. Our review included all eligible trials regardless of type and timing of intervention, and thus, more trials have been included, allowing analysis of some long-term outcomes.

This study had some limitations. There were many fundamental differences between studies, including different sizes and gestational ages of infants at birth and different types and timings of interventions, which could not be explained by our subgroup analyses and are likely to have contributed to the substantial heterogeneity we observed for most outcomes. For this reason, we used random-effect models for all analyses, which allows for differing true effects across studies [85]. In addition, multiple outcomes, multiple time points, and a large number of subgroups were analysed in the current review, which may increase the risk of type 1 error [86]. Despite the large numbers of trials and infants included, the evidence is also limited by the low methodological quality, substantial heterogeneity, and limited data beyond early childhood, making strong conclusions difficult. The quality was low for most of the included trials, largely because of unclear methodology used for random allocation and unclear role of commercial sponsors.

Few studies of nutrition in preterm infants have reported outcomes separately for boys and girls. An individual participant data (IPD) meta-analysis allows more in-depth exploration and more detailed analyses [87]. A planned IPD meta-analysis [88] (PROSPERO CRD42017072683) may prove helpful in further exploring possible sex differences in the effects of macronutrient supplements in infants born small. Further, because of the lack of long-term outcomes, as well as new trials, further followup of existing trials would provide additional critical evidence about the long-term effects of macronutrients on growth of preterm and SGA infants.

Our study suggests that early macronutrient supplements given to infants born small does not alter BMI in childhood. Supplementation may increase weight and length, but not head circumference, in toddlers, but these effects are unlikely to be clinically significant and do not persist in later life. Bone mineralisation is also increased, but only in toddlers. Our analysis does not support concerns from observational studies that early supplement may increase fat mass in later life. However, despite 41 trials with 4,352 infants included, there is still little evidence of the effects of early macronutrient supplements on later growth and body composition.

## Supporting information

S1 Appendix List of planned subgroup analysis. (DOCX)

S2 Appendix List of all references of included studies. (DOCX)

S1 Table Search strategies. (DOCX)

S2 Table Characteristics of included studies. AA, amino acid; AGA, appropriate for gestational age; BMC, bone mineral content; BMD, bone mineral density; BMI, body mass index; BPD, bronchopulmonary dysplasia; BW, birthweight; CA, corrected age; GA, gestational age; HC, head circumference; PMA, postmenstrual age; SGA, small for gestational age.
(DOCX)

S3 Table Macronutrient intakes between trials using formula as primary feed and breastmilk as primary feed. (DOCX)

S4 Table Macronutrient intakes between trials conducted up to 2000 and those conducted after 2000. (DOCX)

S1 Fig Risk of bias. (a) Risk of bias graph: review authors’ judgements about each risk of bias item presented as percentages across all included studies. (b) Risk bias summary: review authors’ judgements about each risk of bias item for each included study.
(TIF)

S2 Fig Sensitivity analyses. Forest plots of effect of macronutrient supplementation on growth outcomes including trials with low risk of bias. *P*-values are from Z test for the summary effect and chi-squared test for heterogeneity. (a) Weight in toddlers (kg), (b) length or height in toddlers (cm), (c) head circumference in toddlers (cm).
(TIF)

S3 Fig Funnel plots. Funnel plot of supplemented versus unsupplemented nutrition for the growth outcomes. (a) Weight in toddlers, (b) length or height in toddlers, (c) head circumference in toddlers. The middle dashed line indicates the overall MD. The dashed line either side represents the pseudo-95% CIs. CI, confidence interval; MD, mean difference.
(TIF)

S4 Fig Forest plots of effect of macronutrient supplementation on growth z-scores. *P*-values are from Z test for the summary effect and chi-squared test for heterogeneity. (a) Weight z-scores, (b) length/ height z-scores, (c) head circumference z-scores.
(TIF)

S5 Fig Forest plots of effect of macronutrient supplementation on skin fold thickness. *P*-values are from Z test for the summary effect and chi-squared test for heterogeneity. (a) Triceps skin fold thickness (mm), (b) subscapular skin fold thickness (mm).
(TIF)

S6 Fig Forest plots of effect of macronutrient supplementation on bone development. *P*-values are from Z test for the summary effect and chi-squared test for heterogeneity. Std.Mean Difference = difference in mean outcome between groups/standard deviation of outcome among participants. (a) BMC, (b) BMD. BMC, bone mineral content; BMD, bone mineral density.
(TIF)

S1 Checklist PRISMA checklist. PRISMA, Preferred Reporting Items for Systematic Reviews and Meta-Analyses.
(DOC)

S1 Protocol Impact of macronutrient supplements on later growth of children born preterm or SGA: Protocol for a systematic review and meta-analysis. SGA, small for gestational age.
(DOCX)

## Abbreviations

BMC: bone mineral content
BMD: bone mineral density
BMI: body mass index
CI: confidence interval
GRADE: Grading of Recommendations Assessment, Development and Evaluation
IPD: individual participant data
MD: mean difference
PRISMA: Preferred Reporting Items for Systematic Reviews and Meta-Analyses
RCT: randomised controlled trial
RR: relative risk
SGA: small for gestational age
SMD: standardised MD
