# Supplementary material for: Impact of macronutrient supplements on later growth of children born preterm or small for gestational age: A systematic review and meta-analysis of randomised and quasirandomised controlled trials
Source: PLoS Med. 2020 May 26;17(5):e1003122. doi: 10.1371/journal.pmed.1003122 (PMC7250404; doi:10.1371/journal.pmed.1003122)
Supplement: S2 Appendix — (DOCX) [file pmed.1003122.s002.docx]

**S2 Appendix. List of all references of included studies**

**Agosti 2003**

1. Agosti M, Vegni C, Calciolari G, Marini A, Gamma Study Group. Post-discharge nutrition of the very low-birthweight infant: interim results of the multicentric GAMMA study. Acta Paediatr Suppl. 2003;91(441):39-43.

**Amesz 2010**

1. Ruys CA, Broring T, van Schie PEM, et al. Neurodevelopment of children born very preterm and/or with a very low birth weight: 8-Year follow-up of a nutritional RCT. Clin Nutr ESPEN. 2019;30:190-198.

2. Ruys CA, van de Lagemaat M, Finken MJ, Lafeber HN. Follow-up of a randomized trial on postdischarge nutrition in preterm-born children at age 8 y. Am J Clin Nutr. 2017;106(2):549-558.

3. van de Lagemaat M, Rotteveel J, Schaafsma A, van Weissenbruch MM, Lafeber HN. Higher vitamin D intake in preterm infants fed an isocaloric, protein- and mineral-enriched postdischarge formula is associated with increased bone accretion. J Nutr. 2013;143(9):1439-1444.

4. van de Lagemaat M, Rotteveel J, van Weissenbruch MM, Lafeber HN. Increased gain in bone mineral content of preterm infants fed an isocaloric, protein-, and mineral-enriched postdischarge formula. Eur J Nutr. 2013;52(7):1781-1785.

5. van de Lagemaat M, Rotteveel J, Muskiet FA, Schaafsma A, Lafeber HN. Post term dietary-induced changes in DHA and AA status relate to gains in weight, length, and head circumference in preterm infants. Prostaglandins Leukot Essent Fatty Acids. 2011;85(6):311-316.

6. Amesz EM, Schaafsma A, Cranendonk A, Lafeber HN. Optimal growth and lower fat mass in preterm infants fed a protein-enriched postdischarge formula. J Pediatr Gastroenterol Nutr. 2010;50(2):200-207.

7. Amesz. Feeding Normo-Caloric Protein Enriched Formula after Term Does Not Affect Growth but Improves Lean Body Mass Gain in Preterm Infants. EPAS2007. 2007;http://www.abstracts2view.com/pas/(5740.8).

8. Amesz. Normocaloric enriched formula after term does not affect growth and body composition in preterm infants. Eur J Pediatr. 2006;165.

**Atkinson 1999**

1. Atkinson SA, Randall-Simpson J, Chang M, Paes B. Randomized trial of feeding nutrient-enriched vs standard formula to premature infants during the first year of life. *Pediatr Res.* 1999;45:276A.

2. Atkinson SA, Paes B, Saigal S, Hussey T, Lee D. Nutrient-enriched discharge formula compared to standard term formula does not benefit growth, bone mineral accretion or trace element status in preterm small for gestational age (SGA) infants to one year corrected age: A RCT. *Pediatr Res.* 2004;55:383A.

**Bellagamba 2016**

1. Bellagamba MP, Carmenati E, D'Ascenzo R, et al. One extra gram of protein to preterm infants from birth to 1800 g: a single-blinded randomized clinical trial. J Pediatr Gastroenterol Nutr. 2016;62(6):879-884.

**Bhatia 1991**

1. Bhatia J, Rassin DK. Feeding the premature infant after hospital discharge: growth and biochemical responses. J Pediatr. 1991;118(4 Pt 1):515-519.

**Brooke 1985**

1. Brooke OG, Kinsey JM. High energy feeding in small for gestation infants. Arch Dis Child. 1985;60(1):42-46.

**Bruton 1998**

1. Brunton JA, Saigal S, Atkinson SA. Growth and body composition in infants with bronchopulmonary dysplasia up to 3 months corrected age: a randomized trial of a high-energy nutrient-enriched formula fed after hospital discharge. J Pediatr. 1998;133(3):340-345.

**Carver 2001**

1. Carver JD, Wu PY, Hall RT, Baggs GE, Blenneman B. Growth of preterm (PT) infants fed similac neocare or similac with iron (SWI) after hospital discharge. Pediatr Res. 1997;41(4 Pt 2):229A.

2. Carver JD, Wu PY, Hall RT, et al. Growth of preterm infants fed nutrient-enriched or term formula after hospital discharge. Pediatrics. 2001;107(4):683-689.

**Chan 1994**

1. Chan GM, Borschel MW, Jacobs JR. Effects of human milk or formula feeding on the growth, behavior, and protein status of preterm infants discharged from the newborn intensive care unit. Am J Clin Nutr. 1994;60(5):710-716.

2. Chan GM. Growth and bone mineral status of discharged very low birth weight infants fed different formulas or human milk. J Pediatr. 1993;123(3):439-443.

**Cooke 2001**

1. Cooke RJ, Embleton ND, Griffin IJ, Wells JC, McCormick KP. Feeding preterm infants after hospital discharge: growth and development at 18 months of age. Pediatr Res. 2001;49(5):719-722.

2. Cooke RJ, Griffin IJ, McCormick K. Adiposity is not altered in preterm infants fed with a nutrient-enriched formula after hospital discharge. Pediatr Res. 2010;67(6):660-664.

3. Cooke RJ, McCormick K, Griffin IJ, et al. Feeding preterm infants after hospital discharge: effect of diet on body composition. Pediatr Res. 1999;46(4):461-464.

4. Cooke RJ, Griffin IJ, McCormick K, et al. Feeding preterm infants after hospital discharge: effect of dietary manipulation on nutrient intake and growth. Pediatr Res. 1998;43(3):355-360.

5. Cooke RJ, Griffin I, Wells J, et al. Formula feeding preterm infants after hospital discharge: 2. Effects on body composition. Pediatr Res. 1996;39(4):306A.

6. Cooke RJ, Griffin I, Wells J, Smith J, Robinson S, Leighton M. Feeding preterm infants after hospital discharge: 1. Effect of type of formula on nutrient intake and growth. Pediatr Res. 1996;39(4):307A.

**Cooper 1985**

1. Cooper PA, Rothberg AD. Feeding of very-low-birth-weight infants with special formula--continued use beyond 2000 g and effects on growth to 1 year. S Afr Med J. 1985;67(18):716-718.

**Cooper 1988**

1. Cooper PA, Rothberg AD, Davies VA, Argent AC. Comparative growth and biochemical response of very low birthweight infants fed own mother's milk, a premature infant formula, or one of two standard formulas. J Pediatr Gastroenterol Nutr. 1985;4(5):786-794.

2. Cooper PA, Rothberg AD, Davies VA. Three year growth and developmental follow up of very low birthweight infants fed own mother's milk (OMM), a premature infant formula (PF) or one of two standard formulas. Pediatr Res. 1988;23:445A.

**Davies 1977**

1. Davies DP. Adequacy of expressed breast milk for early growth of preterm infants. Arch Dis Child. 1977;52(4):296-301.

**DeCurtis 2002**

1. De Curtis M, Pieltain C, Rigo J. Body composition in preterm infants fed standard term or enriched formula after hospital discharge. Eur J Nut. 2002;41(4):177-182.

2. Pieltain C, De Curtis M, Rigo J. In fed formula VLBW infants, is weight gain and weight gain composition related to the protein/energy ratio? J Pediatr Gastroenterol Nutr. 2000;31(Suppl 2):S94.

**Dogra 2017**

1. Dogra S, Thakur A, Garg P, Kler N. Effect of Differential Enteral Protein on Growth and Neurodevelopment in Infants <1500 g: A Randomized Controlled Trial. J Pediatr Gastroenterol Nutr. 2017;64(5):e126-e132.

**Ekcharoen 2015**

1. Ekcharoen C, Tantibhaedhangkul R. Comparing growth rates after hospital discharge of preterm infants fed with either post-discharge formula or high-protein, medium-chain triglyceride containing formula. J Med Assoc Thai. 2015;98(12):1179-1186.

2. Tantibhaedhyangkul R, Ekcharoen C. High-protein, medium-chain triglyceride containing formula as an alternative to post-discharge formula. J Pediatr Gastroenterol Nutr. 2016;63:S113.

**Embleton 2005**

1. Embleton ND, Cooke RJ. Protein requirements in preterm infants: effect of different levels of protein intake on growth and body composition. Pediatr Res. 2005;58(5):855-860.

**Fewtrell 2001**

1. Fewtrell MS, Morley R, Abbott RA, et al. Catch-up growth in small-for-gestational-age term infants: a randomized trial. Am J Clin Nutr. 2001;74(4):516-523.

2. Morley R, Fewtrell MS, Abbott RA, Stephenson T, MacFadyen U, Lucas A. Neurodevelopment in children born small for gestational age: a randomized trial of nutrient-enriched versus standard formula and comparison with a reference breastfed group. Pediatrics. 2004;113(3 Pt 1):515-521.

3. Singhal A, Cole TJ, Fewtrell M, et al. Promotion of faster weight gain in infants born small for gestational age: is there an adverse effect on later blood pressure? Circulation. 2007;115(2):213-220.

4. Singhal A, Kennedy K, Lanigan J, et al. Nutrition in infancy and long-term risk of obesity: evidence from 2 randomized controlled trials. Am J Clin Nutr. 2010;92(5):1133-1144.

**Friel 1993**

1. Friel JK, Andrews WL, Matthew JD, McKim E, French S, Long DR. Improved growth of very low birthweight infants. Nutr Res. 1993;13(6):611-620.

**Jeon 2011**

1. Jeon GW, Jung YJ, Koh SY, et al. Preterm infants fed nutrient-enriched formula until 6 months show improved growth and development. Pediatr Int. 2011;53(5):683-688.

**Koo 2006**

1. Koo WWK, Hockman EM. Posthospital discharge feeding for preterm infants: Effects of standard compared with enriched milk formula on growth, bone mass, and body composition. Am J Clin Nutr. 2006;84(6):1357-1364.

**Lin 2004**

1. Lin YF, Hsieh KS, Chen YY. Nutrient-enriched versus standard term formula feeding in disproportionately small for gestational age infants. Clinical Neonatology. 2004;11(2):36-39.

**Litmanovitz 2007**

1. Litmanovitz I, Eliakim A, Arnon S, et al. Enriched post-discharge formula versus term formula for bone strength in very low birth weight infants: a longitudinal pilot study. J Perinat Med. 2007;35(5):431-435.

2. Litmanovitz I, Dolfin T, Arnon S, et al. Bone strength and growth of preterm infants fed nutrient-enriched or term formula after hospital discharge. Pediatr Res. 2004;55:274A.

**Lucas 1989**

1. Lucas A, Gore SM, Cole TJ, et al. Multicentre trial on feeding low birthweight infants: effects of diet on early growth. Arch Dis Child. 1984;59(8):722-730.

2. Lucas A, Morley R, Cole TJ, et al. Early diet in preterm babies and developmental status in infancy. Arch Dis Child. 1989;64(11):1570-1578.

3. Lucas A, Morley R. Does early nutrition in infants born before term programme later blood pressure? BMJ. 1994;309(6950):304-308.

4. Lucas A, Morley R, Cole TJ, Gore SM. A randomised multicentre study of human milk versus formula and later development in preterm infants. Arch Dis Child Fetal Neonatal Ed. 1994;70(2):F141-146.

5. Bishop NJ, Dahlenburg SL, Fewtrell MS, Morley R, Lucas A. Early diet of preterm infants and bone mineralization at age five years. Acta Paediatr. 1996;85(2):230-236.

6. Fewtrell MS, Prentice A, Jones SC, et al. Bone mineralization and turnover in preterm infants at 8-12 years of age: the effect of early diet. J Bone Miner Res. 1999;14(5):810-820.

7. Morley R, Lucas A. Randomized diet in the neonatal period and growth performance until 7.5-8 y of age in preterm children. Am J Clin Nutr. 2000;71(3):822-828.

8. Singhal A, Cole TJ, Lucas A. Early nutrition in preterm infants and later blood pressure: two cohorts after randomised trials. Lancet. 2001;357(9254):413-419.

9. Singhal A, Farooqi IS, O'Rahilly S, Cole TJ, Fewtrell M, Lucas A. Early nutrition and leptin concentrations in later life. Am J Clin Nutr. 2002;75(6):993-999.

10. Singhal A, Fewtrell M, Cole TJ, Lucas A. Low nutrient intake and early growth for later insulin resistance in adolescents born preterm. Lancet. 2003;361(9363):1089-1097.

11. Singhal A, Cole TJ, Fewtrell M, Lucas A. Breastmilk feeding and lipoprotein profile in adolescents born preterm: follow-up of a prospective randomised study. Lancet. 2004;363(9421):1571-1578.

12. Isaacs EB, Gadian DG, Sabatini S, et al. The effect of early human diet on caudate volumes and IQ. Pediatr Res. 2008;63(3):308-314.

13. Fewtrell MS, Williams JE, Singhal A, Murgatroyd PR, Fuller N, Lucas A. Early diet and peak bone mass: 20 year follow-up of a randomized trial of early diet in infants born preterm. Bone. 2009;45(1):142-149.

14. Lewandowski AJ, Lamata P, Francis JM, et al. Breast Milk Consumption in Preterm Neonates and Cardiac Shape in Adulthood. Pediatrics. 2016;138(1):07.

15. Isaacs EB, Morley R, Lucas A. Early diet and general cognitive outcome at adolescence in children born at or below 30 weeks gestation. J Pediatr. 2009;155(2):229-234.

**26. Lucas 1990**

1. Lucas A, Morley R. Does early nutrition in infants born before term programme later blood pressure? BMJ. 1994;309(6950):304-308.

2. Fewtrell MS, Prentice A, Jones SC, et al. Bone mineralization and turnover in preterm infants at 8-12 years of age: the effect of early diet. J Bone Miner Res. 1999;14(5):810-820.

3. Morley R, Lucas A. Randomized diet in the neonatal period and growth performance until 7.5-8 y of age in preterm children. Am J Clin Nutr. 2000;71(3):822-828.

4. Singhal A, Cole TJ, Lucas A. Early nutrition in preterm infants and later blood pressure: two cohorts after randomised trials. Lancet. 2001;357(9254):413-419.

5. Singhal A, Farooqi IS, O'Rahilly S, Cole TJ, Fewtrell M, Lucas A. Early nutrition and leptin concentrations in later life. Am J Clin Nutr. 2002;75(6):993-999.

6. Singhal A, Fewtrell M, Cole TJ, Lucas A. Low nutrient intake and early growth for later insulin resistance in adolescents born preterm. Lancet. 2003;361(9363):1089-1097.

7. Singhal A, Cole TJ, Fewtrell M, Lucas A. Breastmilk feeding and lipoprotein profile in adolescents born preterm: follow-up of a prospective randomised study. Lancet. 2004;363(9421):1571-1578.

8. Isaacs EB, Gadian DG, Sabatini S, et al. The effect of early human diet on caudate volumes and IQ. Pediatr Res. 2008;63(3):308-314.

9. Fewtrell MS, Williams JE, Singhal A, Murgatroyd PR, Fuller N, Lucas A. Early diet and peak bone mass: 20 year follow-up of a randomized trial of early diet in infants born preterm. Bone. 2009;45(1):142-149.

10. Lewandowski AJ, Lamata P, Francis JM, et al. Breast Milk Consumption in Preterm Neonates and Cardiac Shape in Adulthood. Pediatrics. 2016;138(1):07.

11. Isaacs EB, Morley R, Lucas A. Early diet and general cognitive outcome at adolescence in children born at or below 30 weeks gestation. J Pediatr. 2009;155(2):229-234.

12. Lucas A, Morley R, Cole TJ, et al. Early diet in preterm babies and developmental status at 18 months. Lancet. 1990;335(8704):1477-1481.

13. Lucas A, Morley R, Cole TJ. Randomised trial of early diet in preterm babies and later intelligence quotient. BMJ. 1998;317(7171):1481-1487.

**Lucas 1992**

1. Lucas A, Bishop NJ, King FJ, Cole TJ. Randomised trial of nutrition for preterm infants after discharge. Arch Dis Child. 1992;67(3):324-327.

2. Bishop NJ, King FJ, Lucas A. Increased bone mineral content of preterm infants fed with a nutrient enriched formula after discharge from hospital. Arch Dis Child. 1993;68(5 Spec No):573-578.

**Lucas 1996**

1. Lucas A, Fewtrell MS, Morley R, et al. Randomized outcome trial of human milk fortification and developmental outcome in preterm infants. Am J Clin Nutr. 1996;64(2):142-151.

**Lucas 2001**

1. Lucas A, Fewtrell MS, Morley R, et al. Randomized trial of nutrient-enriched formula versus standard formula for postdischarge preterm infants. Pediatrics. 2001;108(3):703-711.

**Moltu 2013**

1. Moltu SJ, Strommen K, Blakstad EW, et al. Enhanced feeding in very-low-birth-weight infants may cause electrolyte disturbances and septicemia--a randomized, controlled trial. Clin Nutr. 2013;32(2):207-212.

2. Moltu SJ, Sachse D, Blakstad EW, et al. Urinary metabolite profiles in premature infants show early postnatal metabolic adaptation and maturation. Nutrients. 2014;6(5):1913-1930.

3. Moltu SJ, Blakstad EW, Strommen K, et al. Enhanced feeding and diminished postnatal growth failure in very-low-birth-weight infants. J Pediatr Gastroenterol Nutr. 2014;58(3):344-351.

4. Strommen K, Blakstad EW, Moltu SJ, et al. Enhanced nutrient supply to very low birth weight infants is associated with improved white matter maturation and head growth. Neonatology. 2015;107(1):68-75.

5. Blakstad EW, Moltu SJ, Nakstad B, et al. Enhanced nutrition improves growth and increases blood adiponectin concentrations in very low birth weight infants. Food Nutr Res. 2016;60:33171.

6. Strømmen K, Haag A, Moltu SJ, et al. Enhanced nutrient supply to very low birth weight infants is associated with higher blood amino acid concentrations and improved growth. Clin Nutr ESPEN. 2017;18:16-22.

7. Strommen K, Blakstad E, Moltu S, et al. Enhanced Nutrition to Very Low Birth Weight Infants Improves Maturation of Cerebral Connective Tracts. Pediatr Res. 2013.

8. Moltu S, Blakstad E, Strommen K, et al. Enhanced nutritional support enables postnatal growth similar to normal in utero growth in very low birth weight infants. EPAS 2013. 2013.

9. Blakstad EW, Moltu SJ, Nakstad B, et al. Enhanced nutrient supply to very low birth weight (VLBW) infants is associated with improved growth and increased blood adiponectin concentrations. Eur J Pediatr. 2016;175(11):1497-1498.

10. Blakstad EW, Korpela K, Lee S, Nakstad B, Moltu SJ, Strommen K, et al. Enhanced nutrient supply and intestinal microbiota development in very low birth weight infants. Pediatr Res. 2019.

**Mukhopadhyay 2007**

1. Mukhopadhyay K, Mahajan R, Louis D, Narang A. Longitudinal growth of very low birth weight neonates during first year of life and risk factors for malnutrition in a developing country. Acta Paediatr. 2013;102(3):278-281.

2. Mukhopadhyay K, Narnag A, Mahajan R. Effect of human milk fortification in appropriate for gestation and small for gestation preterm babies: a randomized controlled trial. Indian Pediatr. 2007;44(4):286-290.

**O’Connor 2008**

1. Aimone A, Rovet J, Ward W, et al. Growth and body composition of human milk-fed premature infants provided with extra energy and nutrients early after hospital discharge: 1-year follow-up. J Pediatr Gastroenterol Nutr. 2009;49(4):456-466.

2. O'Connor DL, Weishuhn K, Rovet J, et al. Visual development of human milk-fed preterm infants provided with extra energy and nutrients after hospital discharge. JPEN J Parenter Enteral Nutr. 2012;36(3):349-353.

3. Weishuhn K. Visual Development of Premature Infants Fed Human Milk Containing Extra Energy and Nutrients after Hospital Discharge. EPAS 2007. 2007.

4. O'Connor DL, Khan S, Weishuhn K, et al. Growth and nutrient intakes of human milk-fed preterm infants provided with extra energy and nutrients after hospital discharge. Pediatrics. 2008;121(4):766-776.

5. O'Connor. Growth and nutrient intakes of human milk-fed premature infants provided with extra energy and nutrients after hospital discharge. EPAS 2007. 2007.

**Peng 2004**

1. Peng CC, Hsu CH, Kao HA, Hung HY, Chang JH. Feeding with premature or infant formula in premature infants after discharge: comparison of growth and nutrition status. Acta Paediatr Taiwan. 2004;45(3):151-157.

**Pettifor 1989**

1. Pettifor JM, Rajah R, Venter A, et al. Bone mineralization and mineral homeostasis in very low-birth-weight infants fed either human milk or fortified human milk. J Pediatr Gastroenterol Nutr. 1989;8(2):217-224.

**Picaud 2008**

1. Picaud JC, Decullier E, Plan O, et al. Growth and bone mineralization in preterm infants fed preterm formula or standard term formula after discharge. J Pediatr. 2008;153(5):616-621, 621 e611-612.

2. Picaud JC, Plan O, Pidoux O, Reygrobellet BCF, Salle BL, Claris O. Effect of post-discharge nutrition on growth and whole body mineralization in very low birth weight (VLBW) infants. EPAS 2005. 2005.

3. Picaud JC, Plan O, Pidoux O, et al. Effect of post-discharge nutrition on growth and whole-body mineralization in very low birth weight (VLBW) infants. EPAS 2005. 2005.

4. Picaud JC. What formula should we propose for very low birth weight infants after discharge from hospital: preterm formula or standard term formula? *Eur J Pediatr.* 2006;165.

**Roggero 2012**

1. Taroni E, Liotto N, Orsi A, et al. [Quality of post-discharge growth in small for gestational age preterm infants: an explorative study]. Pediatr Med Chir. 2009;31(3):121-125.

2. Roggero P, Giannì ML, Amato O, et al. Growth and fat-free mass gain in preterm infants after discharge: a randomized controlled trial. Pediatrics. 2012;130(5):e1215-1221.

3. Gianni ML, Roggero P, Amato O, et al. Randomized outcome trial of nutrient-enriched formula and neurodevelopment outcome in preterm infants. BMC Pediatr. 2014;14:74.

**Svenningsen 1982**

1. Svenningsen NW, Lindroth M, Lindquist B. Growth in relation to protein intake of low birth weight infants. Early Hum Dev. 1982;6(1):47-58.

2. Svenningsen NW, Lindroth M, Lindquist B. A comparative study of varying protein intake in low birthweight infant feeding. Acta Paediatr Scand Suppl. 1982;296:28-31.

**Tan 2008**

1. Tan MJ, Cooke RW. Improving head growth in very preterm infants--a randomised controlled trial I: neonatal outcomes. Arch Dis Child Fetal Neonatal Ed. 2008;93(5):F337-341.

2. Tan M, Abernethy L, Cooke R. Improving head growth in preterm infants--a randomised controlled trial II: MRI and developmental outcomes in the first year. Arch Dis Child Fetal Neonatal Ed. 2008;93(5):F342-346.

**Wauben 1998**

1. Wauben IP, Atkinson SA, Grad TL, Shah JK, Paes B. Moderate nutrient supplementation of mother's milk for preterm infants supports adequate bone mass and short-term growth: a randomized, controlled trial. Am J Clin Nutr. 1998;67(3):465-472.

2. Wauben IPM, Atkinson SA, Shah JK, Paes B. Growth and body composition of preterm infants: Influence of nutrient fortification of mother's milk in hospital and breastfeeding post-hospital discharge. Acta Paediatr. 1998;87(7):780-785.

3. Wauben I, Gibson R, Atkinson S. Premature infants fed mothers' milk to 6 months corrected age demonstrate adequate growth and zinc status in the first year. Early Hum Dev. 1999;54(2):181-194.

**Wheller 1996**

1. Wheeler RE, Hall RT. Feeding of premature infant formula after hospital discharge of infants weighing less than 1800 grams at birth. J Perinatol. 1996;16(2 Pt 1):111-116.

**Yu 2020**

1. Yu MX, Zhuang SQ, Gao XY, Tong XM, Yue SJ, Shi LP, et al. Effects of a nutrient-dense formula compared with a post-discharge formula on post-discharge growth of preterm very low birth weight infants with extrauterine growth retardation: a multicentre randomised study in China. J Hum Nutr Diet. 2020.

**Zachariassen 2011**

1. Zachariassen G, Faerk J, Grytter C, et al. Nutrient enrichment of mother's milk and growth of very preterm infants after hospital discharge. Pediatrics. 2011;127(4):e995-e1003.

2. Zachariassen G, Faerk J, Esberg BH, et al. Allergic diseases among very preterm infants according to nutrition after hospital discharge. Pediatr Allergy Immunol. 2011;22(5):515-520.

3. Zachariassen G. Nutrition, growth, and allergic diseases among very preterm infants after hospital discharge. Dan Med J. 2013;60(2):B4588.

4. Zachariassen G, Faerk J, Grytter C, et al. Growth among very premature infants fed human milk fortifier while breastfeeding after hospital discharge. Acta Paediatr Suppl. 2009.

5. Toftlund LH, Halken S, Agertoft L, Zachariassen G. Early nutrition and signs of metabolic syndrome at 6 y of age in children born very preterm. Am J Clin Nutr. 2018;107(5):717-724.

6. Toftlund LH, Halken S, Agertoft L, Zachariassen G. Catch-up growth, rapid weight growth, and continuous growth from birth to 6 years of age in very-preterm-born children. Neonatology. 2018;114(4):285-293.

7. Toftlund LH, Agertoft L, Halken S, Zachariassen G. Improved lung function at age 6 in children born very preterm and fed extra protein post-discharge. Pediatr Allergy Immunol. 2019;30(1):47-54.
