# Supplementary material for: Impact of macronutrient supplements on later growth of children born preterm or small for gestational age: A systematic review and meta-analysis of randomised and quasirandomised controlled trials
Source: PLoS Med. 2020 May 26;17(5):e1003122. doi: 10.1371/journal.pmed.1003122 (PMC7250404; doi:10.1371/journal.pmed.1003122)
Supplement: S1 Table — (DOCX) [file pmed.1003122.s003.docx]

**S1 Table. Search strategies**

| **Embase from 1980 to 2019 April 01** | |
| --- | --- |
| # | Search strategies |
| 1 | exp prematurity/ |
| 2 | exp low birth weight/ |
| 3 | exp small for date infant/ |
| 4 | exp very low birth weight/ |
| 5 | (prematur* adj2 infant*).tw. |
| 6 | (prematur* adj2 newborn*).tw. |
| 7 | (prematur* adj2 neonate*).tw. |
| 8 | preterm.tw. |
| 9 | low birth weight.tw. |
| 10 | low birthweight.tw. |
| 11 | VLBW.tw. |
| 12 | LBW.tw. |
| 13 | ELBW.tw. |
| 14 | small for gestation*.tw. |
| 15 | SGA.tw. |
| 16 | (less than adj6 g).tw. |
| 17 | (less than adj3 32 weeks).tw. |
| 18 | birth weight below.tw. |
| 19 | (gestation* adj2 less than).tw. |
| 20 | or/1-19 |
| 21 | exp breast feeding/ |
| 22 | exp infant nutrition/ |
| 23 | exp protein intake/ |
| 24 | exp dietary supplement/ |
| 25 | exp omega 3 fatty acid/ct, ad, dt, ig, pa [Clinical Trial, Drug Administration, Drug Therapy, Intragastric Drug Administration, Parenteral Drug Administration] |
| 26 | exp arachidonic acid/ae, ct, ad, dt, ig, pa, th [Adverse Drug Reaction, Clinical Trial, Drug Administration, Drug Therapy, Intragastric Drug Administration, Parenteral Drug Administration, Therapy] |
| 27 | exp unsaturated fatty acid/ct, dt, pa, th [Clinical Trial, Drug Therapy, Parenteral Drug Administration, Therapy] |
| 28 | exp fat intake/ae, ad, dt [Adverse Drug Reaction, Drug Administration, Drug Therapy] |
| 29 | exp enteric feeding/ |
| 30 | exp parenteral nutrition/ |
| 31 | exp artificial milk/ |
| 32 | exp breast milk/ |
| 33 | exp fortified food/ |
| 34 | exp elemental diet/ |
| 35 | exp baby food/ |
| 36 | (breast milk or human milk).tw. |
| 37 | formula.tw. |
| 38 | PUFA supplement*.tw. |
| 39 | feed* regimen*.tw. |
| 40 | (protein* adj2 concentration*).tw. |
| 41 | probiotic$.tw. |
| 42 | parenteral*.tw. |
| 43 | enteral*.tw. |
| 44 | maternal milk.tw. |
| 45 | multinutrient supplement*.tw. |
| 46 | (breast fed or breastfed).tw. |
| 47 | prebiotic*.tw. |
| 48 | diet* supplement*.tw. |
| 49 | nutrient enriched.tw. |
| 50 | Docosahexaenoic Acid*.tw. |
| 51 | arachidonic acid*.tw. |
| 52 | (glutamine adj2 supplement*).tw. |
| 53 | (taurine adj2 supplement*).tw. |
| 54 | (calcium adj2 supplement*).tw. |
| 55 | palm olein.tw. |
| 56 | palmitic acid.tw. |
| 57 | (fortification or fortified).tw. |
| 58 | fatty acids.tw. |
| 59 | supplement* feed*.tw. |
| 60 | complementary feed*.tw. |
| 61 | nutrition*.tw. |
| 62 | Hydrolysed liquid.tw. |
| 63 | Hydrolyzed liquid.tw. |
| 64 | gamma-linoleic acid.tw. |
| 65 | (diet* adj3 protein*).tw. |
| 66 | or/21-65 |
| 67 | 20 and 66 |
| 68 | Clinical Trial/ |
| 69 | Randomized Controlled Trial/ |
| 70 | exp randomization/ |
| 71 | Single Blind Procedure/ |
| 72 | Double Blind Procedure/ |
| 73 | Crossover Procedure/ |
| 74 | Placebo/ |
| 75 | Randomi?ed controlled trial$.tw. |
| 76 | Rct.tw. |
| 77 | random allocation.tw. |
| 78 | randomly.tw. |
| 79 | randomly allocated.tw. |
| 80 | allocated randomly.tw. |
| 81 | (allocated adj2 random).tw. |
| 82 | Single blind$.tw. |
| 83 | Double blind$.tw. |
| 84 | ((treble or triple) adj blind$).tw. |
| 85 | placebo$.tw. |
| 86 | prospective study/ |
| 87 | or/68-86 |
| 88 | case study/ |
| 89 | case report.tw. |
| 90 | abstract report/ or letter/ |
| 91 | or/88-90 |
| 92 | 87 not 91 |
| 93 | 67 and 92 |
