# Supplementary material for: Impact of macronutrient supplements on later growth of children born preterm or small for gestational age: A systematic review and meta-analysis of randomised and quasirandomised controlled trials
Source: PLoS Med. 2020 May 26;17(5):e1003122. doi: 10.1371/journal.pmed.1003122 (PMC7250404; doi:10.1371/journal.pmed.1003122)
Supplement: S2 Table — AA, amino acid; AGA, appropriate for gestational age; BMC, bone mineral content; BMD, bone mineral density; BMI, body mass index; BPD, bronchopulmonary dysplasia; BW, birthweight; CA, corrected age; GA, gestational age; HC, head circumference; PMA, postmenstrual age; SGA, small for gestational age. (DOCX) [file pmed.1003122.s004.docx]

**S2 Table. Characteristics of included studies**

| **Author/ Year** | **Country** | **Participants** | **Participants, *n*** | **Intervention** | **Control** | **Duration** | **Outcomes** |
| --- | --- | --- | --- | --- | --- | --- | --- |
| Agosti 2003 [1] | Italy | Inclusion criteria: preterm BW < 1,500g and previously fed with a preterm formula  Exclusion criteria: malformations, intraventricular hemorrhage, periventricular leukomalacia, chronic lung disease, necrotising enterocolitis grade >1, total parenteral nutrition >2 weeks, sepsis, retinopathy of prematurity grade >1 | Intervention: 69  Control: 52 | Preterm formula (protein 2.4 g/100 mL, energy 80 kcal/ 100mL) | Standard term formula (protein 1.7 g/100 mL, energy 70 kcal/100 mL) | Started from 40 weeks PMA, stopped at 55 weeks PMA | Weight, length, HC and BMI at 6 and 12 months’ CA (data presented in figures, no standard deviation reported). |
| Amesz 2010 [2] | The Netherlands | Inclusion criteria: preterm GA ≤ 32 weeks or BW ≤ 1,500 g  Exclusion criteria: congenital malformations or conditions known to affect growth or body composition (e.g., severe bronchopulmonary dysplasia, inborn error of metabolism, cardiac or renal disease, necrotizing enterocolitis with substantial gut loss, grade 4 intraventricular hemorrhage) | Intervention: 52  Control:50 | Post-discharge formula (protein 1.7 g/100 mL, fat 3.5 g/ 100mL, carbohydrate 7.0 g/100 mL, energy 67 kcal/100 mL) | Term formula (protein 1.47 g/100 mL, fat 3.5 g/100 mL, carbohydrate 7.2 g/100 mL, energy 70 kcal/100 mL) | Started from term, stopped at 6 months’ CA. | Weight, length, BMI, fat mass, lean mass and BMC, BMD at 6 months’ and 8 years’ CA. |
| Atkinson 1999 [3] | Canada | Inclusion criteria: preterm BW < 1,800g  Exclusion criteria: congenital anomalies or severe lung disease | Intervention: 34 AGA  24 SGA  Control:  36 AGA  29 SGA | Nutrient-enriched formula  (No details of composition) | Standard formula  (No details of composition) | Started from discharge, stopped at 1 year CA | Weight at 9 months’ CA; BMC at 6 months’ CA (Published abstracts). |
| Bellagamba 2016 [4] | Italy | Inclusion criteria: preterm BW 500 to 1249 g. | Intervention: 82  Control: 82 | High protein intake group (protein supplementation started at 1.5 g/kg/day and increased by 0.5 g/kg/day to a maximum of 3.5 g/kg/day on the fifth day after birth) | Standard protein intake group (protein supplementation started at 1.5 g/kg/day and increased by 0.5 g/kg/day to a maximum of 2.5 g/kg/day on the third day after birth) | Started from birth, stopped at discharge. | Weight, length and HC at 2 years’ CA. |
| Bhatia 1991 [5] | USA | Inclusion criteria: BW < 2,000g; body weight < 2,300g at time of hospital discharge; receiving full oral feeding by nipple (100 to 120 kcal/kg per day); parental agreement that their infant would be fed only the study formula as the sole source of nutrition during the entire 4-month period of study.  Exclusion criteria: history of necrotising enterocolitis or with major congenital anomalies, cardiac disease, liver disease, chronic lung disease | Total of 24; number in each group not specified. | Formula 1: (protein 1.5 g/100 mL, fat 3.6 g/100 mL, carbohydrate 7.2 g/100 mL, energy 67 kcal/100 mL) | Formula 2: (protein 1.3 g/100 mL, fat 3.8 g/100 mL, carbohydrate 7.1 g/100 mL, energy 67 kcal/100 mL) | Started 2 to 3 days before discharge, stopped at 16 weeks after discharge. | Weight, length and HC at 1, 2, 3, 4 months after discharge (Data not presented). |
| Biasini 2012 [6] | Italy | Inclusion criteria: preterm BW 580 to 1,250 g and GA < 32 weeks | Intervention: 34  Control:27 | Protein supplemented group (protein 4.8 g/kg/day, energy 141 kcal/day) | Control group (protein 3.5 g/kg/day, energy 135 kcal/day) | Started from the first day of full enteral feeding, stopped at discharge. | Weight, length and HC at 3, 6, 9, 12, 15, 18, 24 months’ CA. |
| Brooke 1985 [7] | UK | Inclusion criteria: term SGA infants with their parents' consent  Exclusion criteria: infants underweight because of wasting | Intervention: 10  Control:7 | High energy formula (protein 2.3 g/100 mL, fat 4.2 g/100 mL, carbohydrate 10.7 g/100 mL, energy 87 kcal/100 mL) | Standard energy formula (protein 1.5 g/100 mL, fat 3.6 g/100 mL, carbohydrate 7.2 g/100 mL, energy 65 kcal/100 mL) | Started from the second week after birth, stopped at 3 months postnatal age. | Weight, length, and HC at 6 weeks and 3 months after birth. |
| Brunton 1998 [8] | Canada | Inclusion criteria: preterm BW < 1,500 g and appropriate for gestational age, had BPD, were formula fed by parental choice, and had not undergone gastrointestinal surgery  Exclusion criterial: major congenital anomalies. | Intervention: 26  Control: 29 | Enriched formula (protein 2.3g/100 mL, energy 90 kcal/100 mL) | Standard isoenergetic formula (protein 1.5 g/100 mL, energy 90 kcal/100 mL) | Started at 37 weeks PMA, stopped at 3 months’ CA. | Weight, length, HC, fat mass and lean mass at 1 and 3 months’ CA. BMC at 3 months’ CA. |
| Carver 2001 [9] | USA | Inclusion criteria: preterm BW < 1,800g or GA < 37 weeks, previous parental decision not to provide breast milk  Exclusion criteria: severe BPD, cardiac, respiratory, gastrointestinal, or other systemic diseases. | Intervention: 67  Control: 56 | Post-discharge formula (protein 1.9 g/100 mL, fat 4.0 g/100 mL, carbohydrate 7.6 g/100 mL, energy 74 kcal/100 mL) | Term formula (protein 1.4 g/100 mL, fat 3.6 g/100 mL, carbohydrate 7.2 g/100 mL energy 67 kcal/100 mL) | Started 2 to 4 days before discharge, stopped at 12 months' CA. | Weight, length, and HC at 1, 2, 3, 6, 9 and 12 months’ CA. |
| Chan 1994 [10] | USA | Inclusion criteria: preterm BW < 1,650 g and weight at hospital discharge ≥ 1,800 g  Exclusion criteria: necrotising enterocolitis, congenital anomalies, hepatic disease, cardiac disease, BPD. | Preterm formula: 14  Low birth weight formula: 14  Standard formula: 15 | Preterm formula (protein 1.85-1.94 g/100 mL, fat 3.75-3.83 g/100 mL, carbohydrate 7.1-7.2 g/100 mL, energy 69.4-71.0 kcal/100 mL);  Low birth weight formula (protein 1.71-1.78 g/100 mL, fat 3.62-3.81 g/100 mL, carbohydrate 6.8-7.1g/100 mL, energy 66.7-66.9 kcal/100 mL) | Standard formula (protein 1.52-1.54 g/100 mL, fat 3.64-3.74 g/100 mL, carbohydrate 7.1-7.3 g/100 mL, energy 67.4-68.9 kcal/100 mL) | Started from discharge, stopped 8 weeks after discharge. | Weight and BMC at 2 weeks, 8 weeks and 16 weeks after discharge. |
| Cooke 2001 [11] | UK | Inclusion criteria: preterm GA ≤ 34 weeks and BW ≤ 1,750 g, growing normally at the time of hospital discharge, i.e. ≥25 g/d Exclusion criteria: systemic disease or requiring medication. | Intervention: 56  Control: 57 | Preterm formula (protein 2.2 g/100 mL, fat 4.4 g/100 mL, carbohydrate 8.5 g/100 mL, energy 80 kcal/100 mL) | Term formula (protein 1.4 g/100 mL, fat 3.6 g/100 mL, carbohydrate 7.5 g/100 mL, energy 66 kcal/100 mL) | Started from discharge, stopped at 6 months’ CA. | Weight, length, and HC at 3, 6, 9, 12, 18 months’ CA. Fat mass, BMC, BMD at 3, 6 and12 months’ CA. |
| Cooper 1985 [12] | South Africa | Inclusion criteria: preterm BW 1,200 to 1,500 g and GA < 36 weeks Exclusion criteria: needing ventilator support | Intervention: 4  Control:4 | Preterm formula (protein 1.94 g/100 mL, fat 3.4 g/100 mL, carbohydrate 7.3 g/100 mL, energy 67 kcal/100 mL) | Standard formula (protein 1.5 g/100 mL, fat 1.6 g/100 mL, carbohydrate 7.2 g/100 mL, energy 67 kcal/100 mL) | Started when reached a weight of 2,000 g, stopped after 8 weeks. | Weight, length, and HC at 3 months’ CA. |
| Cooper 1988 [13] | South Africa | Inclusion criteria: preterm BW < 1,600 g  Exclusion criteria: major congenital abnormalities, congenital infections, or severe intrauterine growth retardation. Withdrawn if intake > 100 kcal/kg/ day from mother’s own milk. | Intervention: 10  Control: 10 | Preterm formula (protein 2.2 g/100 mL, fat 3.7 g/100 mL, carbohydrate 8.6 g/100 mL, energy 75 kcal/100 mL) | Standard formula (protein 1.6 g/100 mL, fat 3.4 g/100 mL, carbohydrate 7.4 g/100 mL, energy 67 kcal/100 mL) | Started when half caloric intake was via the enteral route, stopped after 5 weeks. | Weight, length and HC at 1 and 3 years. Skinfold thickness at 1 year. |
| Davies 1977 [14] | UK | Inclusion criteria: preterm GA 28 to 36 weeks  Exclusion criteria: Multiple births, major congenital abnormalities, chromosome disorders, and congenital infections | Intervention: 34  Control: 34 | Ostermilk Formula (protein 2.7 g/100 mL, fat 3.28 g/100 mL, carbohydrate 8.4 g/100 mL, energy 70 kcal/100 mL) | Mature breast milk (protein 1.1 g/100 mL, fat 4.4 g/100 mL, carbohydrate 7.3 g/100 mL, energy 71 kcal/100 mL) | Started from the first day after birth, stopped at 2 months after birth. | Weight, length and HC at 1 and 2 months. |
| De Curtis 2002 [15] | Italy | Inclusion criteria: preterm BW < 1,750 g and GA<35 weeks  Exclusion criteria: infants with clinical problems | Intervention: 16  Control: 17 | Post discharge formula (protein 1.8g/100 mL, fat 4.1g/100 mL, carbohydrate 7.5 g/100 mL, energy 74 kcal /100 mL) | Term formula (protein 1.4 g/100 mL, fat 3.6 g/100 mL, carbohydrate 7.1 g/100 mL, energy 66 kcal/100 mL) | Started from start of enteral feeds, stopped after 2 months. | Weight, length, and HC, fat mass, lean mass and BMC at 1 month and 2 months. |
| Dogra 2017 [16] | India | Inclusion criteria: preterm BW < 1,500 g or GA < 32 weeks and reached a feed volume of 100 mL/kg/day  Exclusion criteria: lethal congenital malformations. | Intervention: 59  Control: 56 | Fortified breast milk with higher protein intake (fortifier contained protein 1.0 g/100 mL, fat 0.01 g/100 mL, carbohydrate 3.6 g/100 mL, energy 17.2 kcal/100 mL) | Fortified breast milk with standard protein intake (fortifier contained protein 0.4 g/100 mL, fat 0.2 g/100 mL, carbohydrate 2.4 g/100 mL; energy 13 kcal/100 mL) | Started when reached a feed volume of 100 mL/kg/day, stopped at discharge or when totally directly breast-fed, whichever was earlier. | Weight, length and HC at 12 to 18 months’ CA. |
| Ekcharoen 2015 [17] | Thailand | Inclusion criteria: preterm GA 35 to 36 weeks and weight 1800 to 3000g at hospital discharge  Exclusion criteria: congenital anomalies, known chromosomal abnormalities, gastro-intestinal problems that limited feeding, cow milk protein allergy. | Intervention: 6  Control: 5 | Post discharge formula (protein 1.9/100mL, fat 4.1 g/100 mL, carbohydrate 7.5 g/100 mL, energy 74.4 kcal /100 mL) | High-protein, medium chain triglyceride formula (protein 2.0/100 mL, fat 3.4 g/100 mL, carbohydrate 7.3 g/100 mL, energy 67.7 kcal/100 mL) | Started at discharge, stopped when weight at least 3,000 g or at 40 weeks PMA. | Weight gain, length gain and HC gain at 28, 56 and 84 days after hospital discharge. |
| Embleton 2005 [18] | UK | Inclusion criteria: preterm GA ≤ 34 weeks and BW ≤ 1,750g, tolerating enteral intake ≥ 150 mL/kg/d for ≥ 48 h and current weight ≥ 1,000 g. | Formula A: 25  Formula B: 25  Formula C: 26 | Formula A (protein 2.6 g/100 mL, fat 4.3g/100 mL, carbohydrate 7.9g/100 mL, energy 80 kcal/100 mL);  Formula B (protein 2.4 g/100 mL, fat 4.4g/100 mL, carbohydrate 7.9g/100 mL, energy 80kcal/100 mL) | Formula C (protein 2.2 g/100 mL, fat 4.5 g/100 mL, carbohydrate 7.9 g/100 mL, energy 80 kcal/100 mL) | Started when full enteral feeding 150 mL/kg/d, stopped at 12 week’ CA. | Weight, length, HC, fat mass, lean mass, BMC and BMD at 3 months’ CA. |
| Fewtrell 2001 [19] | UK | Inclusion criteria: term GA ≥ 37 weeks and BW < 10th centile for gestation and sex (UK growth charts). | Intervention: 152  Control: 147 | Enriched formula (protein 1.9 g/100 mL, fat 4.0 g/100 mL, carbohydrate 7.2 g/100 mL, energy 72 kcal/100 mL) | Term formula (protein 1.5 g/100 mL, fat 3.9 g/100 mL, carbohydrate 7.0 g/100 mL, energy 68 kcal/100 mL) | Started within the first week, stopped at 9 months’ CA. | Weight, length, HC at 9 and 18 months’, and at 6 to 8 years’ CA; fat mass and fat free mass at 6 to 8 years’ CA. |
| Friel 1993 [20] | Canada | Inclusion criteria: preterm BW < 1,500g.  Exclusion criteria: breast-fed, hydrocephalus, liver dysfunction or congenital malformations. | Intervention: 27  Control: 27 | Low birthweight formula (protein 1.7 g/100 mL, fat 3.7g/100 mL, carbohydrate 7.1g/100 mL, energy 67 kcal/100 mL) | Regular term formula  (protein 1.6 g/100 mL, fat 3.6 g/100 mL, carbohydrate 7.3g/100 mL, energy 67 kcal/100 mL) | Started when reached 1,850 g, stopped at 5 months after discharge. | Weight, length, and HC gain over the study period. |
| Jeon 2011 [21] | Korea | Inclusion criteria: preterm GA < 33 weeks and BW < 1,500 g, formula as the primary food source  Exclusion criteria: chromosomal disorders, serious congenital malformations at discharge that would affect growth and development. | Intervention: 35  Control: 34 | Preterm formula (protein 2.3 g/100 mL, fat 4.1 g/100 mL, carbohydrate 8.5 g/100 mL, energy 80 kcal/100 mL) | Term formula (protein 1.6 g/100 mL, fat 3.5 g/100 mL, carbohydrate 7.2 g/100 mL, energy 67 kcal/100 mL) | Started at term, stopped at 6 months’ CA. | Weight, length, and HC at 3, 6, 9, 12, 15, and 18 months’ CA. |
| Koo 2006 [22] | USA | Inclusion criteria: preterm GA ≤ 34 weeks and BW 630 to 1,620 g, intact gastrointestinal tract, tolerated full enteral feeding, expected soon to be ready for hospital discharge. | Intervention: 44  Control: 45 | Nutrient-enriched formula (protein 1.9 g/100 mL, fat 4.1 g/100 mL, carbohydrate 7.6 g/100 mL, energy 74 kcal/100 mL) | Term formula (protein 1.4 g/100 mL, fat 3.6 g/100 Ml, carbohydrate 7.2 g/100 mL, and energy 67 kcal/100 mL) | Started at discharge, stopped at 12 months after discharge. | Weight, length, and HC at 2, 4, 6, 9, 12 months after discharge. Fat mass, lean mass and BMC at 2, 4, 6, 12 months after discharge. |
| Lin 2004 [23] | China | Inclusion criteria: term GA ≥ 37 weeks and birth weight < 10th centile.  Exclusion criteria: severe medical problems or breast-fed. | Intervention: 20  Control: 20 | Nutrient-enriched formula (protein 1.9 g/100 mL, fat 4.1 g/100 mL, carbohydrate 7.7 g/100 mL, energy 74 kcal/100 mL) | Term formula (protein 1.4 g/100 mL, fat 3.7 g/100 mL, carbohydrate 7.1 g/100 mL, energy 68 kcal/100 mL) | Started at discharge, stopped at 3 months after discharge. | Weight, length, HC at 1, 2, and 3 months. |
| Litmanovitz 2007 [24] | Israel | Inclusion criteria: preterm BW < 1,500 g and appropriate for gestational age, formula fed  Exclusion criteria: severe central nervous system disorder, major congenital anomalies, chronic lung disease, prior diagnosis of necrotising enterocoliti | Intervention: 10  Control: 10 | Enriched post-discharge formula (protein 1.9g/100mL, energy 74 kcal/100mL) | Term formula (protein 1.5 g/100mL, energy 67 kcal/100mL) | Started at term (discharge), stopped at 6 months’ CA. | Weight, length, and HC at 3 and 6 months’ CA. |
| Lucas 1989 [25] | UK | Inclusion criteria: preterm GA < 37 weeks and BW < 1,850g.  Exclusion criteria: major congenital abnormality known to impair growth or development, died before randomisation within the first 48 hours | (1) Lucas 1989a Intervention: 76  Control: 83 | (1) Lucas 1989a: Preterm formula as sole diet (protein 2.0 g/100 mL, fat 4.9 g/100 mL, carbohydrate 7.0 g/100 mL, energy 80 kcal/100 mL) | (1) Lucas 1989a: Banked breast milk as sole diet (protein 1.1 g/100 mL, fat 1.7 g/100 mL, carbohydrate 7.1 g/100 mL, energy 46 kcal/100 mL) | Started within 48 hours, stopped at discharge or reached 2,000 g. | Weight, length and HC at 9, 18 months’ and 7.5 to 8 years’, 8 to 12 years’, 13 to 16 years’ and 20 years’ CA. BMD and BMC at 8 to 12 years and 20 years CA. |
|  |  |  | (2) Lucas 1989b:  Intervention: 173  Control: 170 | (2) Lucas 1989b  Preterm formula as supplement | (2) Trial b:  Banked breast milk as supplement; |  |  |
|  |  |  | (3) Lucas 1989c: combined Lucas 1989a and Lucas | (3) Lucas 1989c: combined Lucas 1989a and Lucas 1989b | (3) Lucas 1989c: combined Lucas 1989a and Lucas 1989b |  |  |
| Lucas 1990 [26] | UK | Inclusion criteria: preterm GA < 37 weeks and BW < 1,850g  Exclusion criteria: major congenital abnormality known to impair growth or development, died before randomisation within the first 48 hours | (1) Lucas 1990a:  Intervention: 81  Control: 79 | (1) Lucas 1990a:  Preterm formula as sole diet (protein 2.0 g/100 mL, fat 4.9 g/100 mL, carbohydrate 7.0 g/100 mL, energy 80 kcal/100 mL) | (1) Lucas 1990a:  Term formula as sole diet (protein 1.5 g/100 mL, fat 3.8 g/100 mL, carbohydrate 7.0 g/100 mL, energy 68 kcal/100 mL) | Started within 48 hours, stopped at discharge or reached 2,000 g | Weight, length and HC at 9, 18 months’ and 7.5 to 8 years’, 8 to 12 years’, 13 to 16 years’ and 20 years’ CA.  BMC and BMD at 8 to 12 years’ and 20 years’ CA. |
|  |  |  | (2) Lucas 1990b:Intervention: 132  Control: 132 | (2) Lucas 1990b  Preterm formula as supplement | (2) Lucas 1990b: Term formula as supplement |  |  |
|  |  |  | (3) Lucas 1990c: Combined Lucas 1990a and Lucas 1990b:  Intervention: 213  Control: 211 | (3) Lucas 1990c: Combined Lucas 1990a and Lucas 1990b | (3) Lucas 1990c: Combined Lucas 1990a and Lucas 1990b |  |  |
| Lucas 1992 [27] | UK | Inclusion criteria: preterm BW < 1,850g and GA < 37 weeks, formula fed during hospital stay  Exclusion criteria: congenital malformations and disease likely to influence growth and neurodevelopment | Intervention: 16  Control: 15 | Post-discharge formula (protein 1.9 g/100 mL, fat 4.0 g/100 mL, carbohydrate 7.3 g/100 mL, energy 72 kcal/100 mL) | Term formula (protein 1.5 g/100 mL, energy 67 kcal/100 mL, fat and carbohydrate not specified) | Started before discharge, stopped at 9 months’ CA. | Weight, length, HC and BMC at 3 and 9 months’ CA. |
| Lucas 1996 [28] | UK | Inclusion criteria: preterm BW < 1,850 g and GA <37 weeks, survived to be assigned to a study group between 48 and 72 hours of age  Exclusion criteria: major congenital anomalies | Intervention: 137  Control: 138 | Fortified human breast milk (fortifier contained protein 0.7g/100 mL, fat 0.05g/100 mL, carbohydrate 2.73g/100 mL, energy 14kcal/100 mL) | Human breast milk | Started within 48 hours, stopped at discharge or reached 2,000 g. | Weight, length and HC at 9, 18 months’ CA. |
| Lucas 2001 [29] | UK | Inclusion criteria: preterm GA<37 weeks and BW< 1,750g  Exclusion criteria: congenital malformations or conditions known to affect growth or development. | Intervention: 113  Control: 116 | Post-discharge formula (protein 1.9 g/100 mL, fat 4.0 g/100 mL, carbohydrate 7.2 g/100 mL, energy 72 kcal/100 mL) | Term formula (protein 1.5 g/100 mL, fat 3.8 g/100 mL, carbohydrate 7.0 g/100 mL, energy 68 kcal/100 mL) | Started one week before discharge, stopped at 9 months CA. | Weight, length, HC at 9 and 18 months’ CA. |
| Moltu 2013 [30] | Norway | Inclusion criteria: preterm GA < 37 weeks and BW < 1,500g  Exclusion criteria: congenital malformations, chromosomal abnormalities, critical illness with short life expectancy, clinical syndromes known to affect growth and development. | Intervention: 24  Control: 24 | Enhanced nutrient: Parenteral nutrition: started with 3.5 g/kg/d AA.  Full enteral feeding: intervention group received 10% higher energy and 20% higher protein than control group. | Standard nutrient:  Parenteral nutrition: started with 2.0 g/kg/d AA | Started within 24 hours after birth, stopped at 52 weeks PMA or when reached 5.5kg | Weight z-score at 2, 5, 12 and 24 months; length z-score, HC z-score at 6, 12, 24 months |
| Mukhopadhyay 2007 [31] | India | Inclusion criteria: preterm GA ≤ 34 weeks and BW ≤ 1,500g, reached feed volume of 150 mL/kg/day, feed constituted at least 80% breast milk  Exclusion criteria: major congenital malformation, gastrointestinal abnormalities. | Intervention: 85  Control: 81 | Fortified human milk: (fortifier contained protein 0.4 g/100 mL; fat 0.2 g/100 mL; carbohydrate 2.4 g/100 mL; energy 13 kcal/100 mL) | Exclusive human milk. | Started when feed volume reached 150 ml/kg/day, stopped when reached 2 kg or full breastfeeds. | Weight, weight z-score, length, length z-score, HC and HC z-score at 12 months’ CA. |
| O’Connor 2008 [32] | Canada | Inclusion criteria: preterm GA < 33 weeks, BW 750 to 1,800 g, received ≥ 80% of their total feedings as human milk 3 days before hospital discharge  Exclusion criteria: serious congenital or chromosomal anomalies that could affect growth, grade 3 or 4 periventricular or intraventricular haemorrhage, oral steroids within 14 days of randomisation, severe asphyxia, known maternal alcohol or drug abuse | Intervention: 19  Control: 20 | Human milk with multinutrient fortifier (protein 2.0 g/100 mL, fat 4.2 g/100 mL, carbohydrate 8.8 g/100 mL, energy 81 kcal/100 mL) | Unfortified human milk (protein 1.3 g/100 mL, fat 3.9 g/100 mL, carbohydrate 7.2g/100 mL, energy 68 kcal/100 mL) | Started from discharge, stopped at 12 weeks after discharge. | Weight, length, HC at 4, 8, 12 weeks after discharge and 4, 6 and 12 months’ CA. Fat mass and lean mass at 4 and 12 months’ CA. BMC and BMD at 4 and 12 months’ CA. |
| Peng 2004 [33] | China | Inclusion criteria: GA ≤ 35 weeks, BW ≤ 1,850g, growing normally at the time of hospital discharge  Exclusion criteria: evidence of systemic diseases, medication requirement, congenital anomaly. | Intervention: 19  Control: 15 | Premature infant formula (protein 2.4 g/100 mL, fat 4.1 g/100 mL, carbohydrate 9.0 g/100 mL, energy 81 kcal/100 mL) | Standard term formula (protein: 1.4 g/100 mL, fat 3.7 g/100 mL, carbohydrate 7.1 g/100 mL, energy 67.6 kcal/100 mL) | Started after hospital discharge, stopped at 6 months’ corrected age. | Weight, length, HC at 1, 2, 3, 4, 5, 6 months’ corrected age. |
| Pettifor 1989 [34] | South Africa | Inclusion criteria: BW 1,000 to 1,500 g  Exclusion criteria: major congential abnormalities, metabolic disturbances, serious infections, required ventilator support | Intervention: 29  Control: 30 | Fortified breast milk (protein 2.2 g/100 mL, fat 4.2 g/100 mL, carbohydrate 7.6 g/100 mL, energy 74.6 kcal/100 mL) | Breast milk (protein 2.1 g/100 mL, fat 3.9g/100 mL, carbohydrate 6.5 g/100 mL, energy 69.2 kcal/100 mL) | Started on day four after birth, stopped when reached 1,800g | BMC and BMD at 3 months after birth. |
| Picaud 2008 [35] | France | Inclusion criteria: GA ≤ 33 weeks, BW < 1,750 g.  Exclusion criteria: major congenital malformations | Intervention: 23  Control: 26 | Preterm formula (protein 2.3 g/100 mL, fat 4.2 g/100 mL, carbohydrate 8.5 g/100 mL, energy 81 kcal/100 mL) | Term formula (protein 1.7 g/100 mL, fat 3.2 g/100 mL, carbohydrate 7.85 g/100 mL, energy 67 kcal/100 mL) | Started after discharge, stopped 2 months after discharge. | Weight, length, HC at 2, 4 and 12 months’ CA. BMI at 12 months’ CA. Fat mass at 2 and 4 months’ CA. BMC and BMD at 2 and 4 months’ CA |
| Roggero 2012 [36] | Italy | Inclusion criteria: GA ≤ 32 weeks, BW ≤ 1,500g, being fed human milk for < 20% of total milk intake  Exclusion criteria: congenital malformations, conditions that interfere with growth or body composition | Intervention: 110  Control: 107 | Nutrient-enriched formula (protein 2.0 g/100 mL, fat 4.1 g/100 mL, carbohydrate 7.5 g/100 mL, energy 75 kcal/100 mL) | Term formula (protein 1.4g/100 mL, fat 3.7 g/100 mL, carbohydrate 7.4 g/100 mL, energy 68 kcal/100 mL) | Started from term CA, stopped at 6 months. | Weight, length, HC at 1, 3, 6 and 24 months’ CA. Body composition at 1, 3, 6 month’ CA. |
| Svenningsen 1982 [37] | Sweden | Inclusion criteria: Very low birth weight preterm infants with mean BW 1385 ± 343 g and GA 30.8 ± 2.9 weeks. | Intervention: 16  Control: 14 | Nutrition enriched formula (protein 2.1 g/100 mL, energy 69.5 kcal/100 mL) | Standard formula (protein 1.6 g/100 mL, energy 68.5 kcal/100 mL) | Started from the third week after birth, stopped at the seventh week after birth. | Weight, length, and HC at 5, 8, 12, 18 and 24 months. |
| Tan 2008 [38] | UK | Inclusion criteria: GA < 29 weeks.  Exclusion criteria: Triplets and higher multiplicity, admitted after 7 days of age, major congenital abnormalities | Intervention: 68  Control: 74 | Parenteral intake with protein 4 g/kg/day, fat 4 g/kg/day, carbohydrate 16.3 g/kg/day, energy 117 kcal/kg/day; enteral intake breast milk or formula with target protein 4 g/kg/day, energy 133-150 kcal/kg/day | Parenteral intake with protein 3 g/kg/day, fat 3g/kg/day, carbohydrate 13.5 g/kg/day, energy 93 kcal/kg/day; enteral intake breast milk or formula with target protein 3.3 g/kg/day, energy 133 kcal/kg/day | Started when infants received parenteral and enteral nutrition from the first week, stopped at 34 weeks’ PMA. | Weight, length, HC at 3 and 9 months’ CA. |
| Yu 2020 [39] | China | Inlcusion criteria: BW< 1,500g, GA < 37 weeks, mother could not provide breask milk and fed a preterm formula during the hospital stay, body weight ≤ 10^th^ percentile of intrauterine growth expectation based on estimated GA at the time of hospital discharge, and fed orally at the time of hospital discharge;  Exclusion criteria: congenital malformations, metabolic disease, and/or major gastrointestinal, hepatic or renal dysfunction; cow milk allergy; did not complete before body weight reached the 50^th^ percentile of interauterine growth expectation based on estimated GA or ≤ 6 months of follow-up evaluation. | Intervention: 24;  Control: 24 | Nutrient-dense formula (protein 2.6 g/100 ml, fat 5.4 g/100 ml, carbohydrate 9.9 g/100 ml, energy 100 kcal/ 100ml) | Post-discharge formula (protein 1.95 g/100 ml, fat 4.09 g/100 ml, carbohydrate 7.23 g/100 ml, energy 74 kcal/ 100ml) | Started after discharge, stopped 6 months’ CA. | Weight, length, HC at 1, 2, 3, 4, 5 and 6 months’ CA. |
| Wauben 1998 [40] | Canada | Inclusion criteria: BW < 1,800g, appropriate weight for gestational age, postnatal age > 1 week, full oral foods (no parenteral nutrition and > 160 mL/kg/d enterally) tolerated for ≥ 5 days, weight gain > 10 g/kg/d  Exclusion criteria: severe congenital malformation, chromosomal abnormalities, gastrointestinal disease. | Intervention: 12  Control: 13 | Mother's milk with multinutrient fortifier (fortifier protein 0.4 g/100 mL, fat 34.7g/100 mL) | Mother’s milk supplemented with calcium and phosphorus | Started when expressed mother’s milk > 80% of total enteral intake, stopped when discharged if PMA> 38 weeks | Weight, length, and HC and body composition at 3, 6, and 12 months’ CA. Bone mineral content and protein and energy intake at 3, 6, 12 months’ CA. |
| Wheeler 1996 [41] | USA | Inclusion criteria: preterm GA < 35 weeks and BW < 1,800g; normally grown in utero, free of medical conditions that affect nutrition and growth  Exclusion criteria: necrotising enterocolitis, chronic lung disease (oxygen need after hospital discharge), central nervous system disease, other conditions affecting nutrient intake or anthropometrics | Intervention: 23  Control: 20 | Whey predominant premature infant formula (protein 1.8 g/100 mL, fat 3.7 g/100 mL, carbohydrate 7.2 g/100 mL, energy 68 kcal/100 mL) | Standard formula (protein 1.5 g/100 mL, fat 3.6 g/100 mL, carbohydrate 7.2 g/100 mL, energy 68 kcal/100 mL) | Started 1 week before hospital discharge, stopped 8 weeks after discharge | Weight, length and HC 12 weeks after hospital discharge. |
| Zachariassen 2001 [42] | Denmark | Inclusion criteria: preterm GA ≤32 weeks, breastfeeding  Exclusion criteria: severe diseases, circumstances influencing eating and feeding ability at discharge. | Intervention: 105  Control: 102 | Fortified mother's milk. Component of fortifier: (protein 1.4 g/day, energy 17.5 kcal/day) | Unfortified mother's milk | Started shortly before discharge, stopped at 4 months’ CA. | Weight, length and HC at 4, 6, 12 months and 6 years’ CA. Body composition at 6 years’ CA. |

References：

1. Agosti M, Vegni C, Calciolari G, Marini A, Gamma Study Group. Post-discharge nutrition of the very low-birthweight infant: interim results of the multicentric GAMMA study. Acta Paediatr Suppl. 2003;91(441):39-43.

2. Amesz EM, Schaafsma A, Cranendonk A, Lafeber HN. Optimal growth and lower fat mass in preterm infants fed a protein-enriched postdischarge formula. J Pediatr Gastroenterol Nutr. 2010;50(2):200-7.

3. Atkinson SA, Randall-Simpson J, Chang M, Paes B. Randomized trial of feeding nutrient-enriched vs standard formula to premature infants during the first year of life. Pediatr Res. 1999;45:276.

4. Bellagamba MP, Carmenati E, D'Ascenzo R, Malatesta M, Spagnoli C, Biagetti C, et al. One extra gram of protein to preterm infants from birth to 1800 g: a single-blinded randomized clinical trial. J Pediatr Gastroenterol Nutr. 2016;62(6):879-84.

5. Bhatia J, Rassin DK. Feeding the premature infant after hospital discharge: growth and biochemical responses. J Pediatr. 1991;118(4 Pt 1):515-9.

6. Biasini A, Marvulli L, Neri E, China M, Stella M, Monti F. Growth and neurological outcome in ELBW preterms fed with human milk and extra-protein supplementation as routine practice: do we need further evidence? J Matern Fetal Neonatal Med. 2012;25 Suppl 4:72-4.

7. Brooke OG, Kinsey JM. High energy feeding in small for gestation infants. Arch Dis Child. 1985;60(1):42-6.

8. Brunton JA, Saigal S, Atkinson SA. Growth and body composition in infants with bronchopulmonary dysplasia up to 3 months corrected age: a randomized trial of a high-energy nutrient-enriched formula fed after hospital discharge. J Pediat. 1998;133(3):340-5.

9. Carver JD, Wu PY, Hall RT, Ziegler EE, Sosa R, Jacobs J, et al. Growth of preterm infants fed nutrient-enriched or term formula after hospital discharge. Pediatrics. 2001;107(4):683-9.

10. Chan GM, Borschel MW, Jacobs JR. Effects of human milk or formula feeding on the growth, behavior, and protein status of preterm infants discharged from the newborn intensive care unit. Am J Clin Nutr. 1994;60(5):710-6.

11. Cooke RJ, Embleton ND, Griffin IJ, Wells JC, McCormick KP. Feeding preterm infants after hospital discharge: growth and development at 18 months of age. Pediatr Res. 2001;49(5):719-22.

12. Cooper PA, Rothberg AD. Feeding of very-low-birth-weight infants with special formula--continued use beyond 2000 g and effects on growth to 1 year. S Afr Med J. 1985;67(18):716-8.

13. Cooper PA, Rothberg AD, Davies VA. Three year growth and developmental follow up of very low birthweight infants fed own mother's milk (OMM), a premature infant formula (PF) or one of two standard formulas. Pediatr Res. 1988;23:445A.

14. Davies DP. Adequacy of expressed breast milk for early growth of preterm infants. Arch Dis Child. 1977;52(4):296-301.

15. De Curtis M, Pieltain C, Rigo J. Body composition in preterm infants fed standard term or enriched formula after hospital discharge. Eur J Nutr. 2002;41(4):177-82.

16. Dogra S, Thakur A, Garg P, Kler N. Effect of differential enteral protein on growth and nurodevelopment in infants <1500 g: a randomized controlled trial. J Pediatr. 2017;64(5):e126-e32.

17. Ekcharoen C, Tantibhaedhyangkul R. Comparing Growth Rates after Hospital Discharge of Preterm Infants Fed with Either Post-Discharge Formula or High-Protein, Medium-Chain Triglyceride Containing Formula. J Med Assoc Thai. 2015;98(12):1179-86.

18. Embleton ND, Cooke RJ. Protein requirements in preterm infants: effect of different levels of protein intake on growth and body composition. Pediatr Res. 2005;58(5):855-60.

19. Fewtrell MS, Morley R, Abbott RA, Singhal A, Stephenson T, MacFadyen UM, et al. Catch-up growth in small-for-gestational-age term infants: a randomized trial. Am J Clin Nutr. 2001;74(4):516-23.

20. Friel JK, Andrews WL, Matthew JD, McKim E, French S, Long DR. Improved growth of very low birthweight infants. Nutr Res. 1993;13(6):611-20.

21. Jeon GW, Jung YJ, Koh SY, Lee YK, Kim KA, Shin SM, et al. Preterm infants fed nutrient-enriched formula until 6 months show improved growth and development. Pediatr Int. 2011;53(5):683-8.

22. Koo WW, Hockman EM. Posthospital discharge feeding for preterm infants: effects of standard compared with enriched milk formula on growth, bone mass, and body composition. Am J Clin Nutr. 2006;84(6):1357-64.

23. Lin YF, Hsieh KS, Chen YY. Nutrient-enriched versus standard term formula feeding in disproportionately small for gestational age infants. Clin Neonatology. 2004;11(2):36-9.

24. Litmanovitz I, Eliakim A, Arnon S, Regev R, Bauer S, Shainkin-Kestenbaum R, et al. Enriched post-discharge formula versus term formula for bone strength in very low birth weight infants: a longitudinal pilot study. J Perinat Med. 2007;35(5):431-5.

25. Lucas A, Morley R, Cole TJ, Gore SM, Davis JA, Bamford MF, et al. Early diet in preterm babies and developmental status in infancy. Arch Dis Child. 1989;64(11):1570-8.

26. Lucas A, Morley R, Cole TJ, Gore SM, Lucas PJ, Crowle P, et al. Early diet in preterm babies and developmental status at 18 months. Lancet. 1990;335(8704):1477-81.

27. Lucas A, Bishop NJ, King FJ, Cole TJ. Randomised trial of nutrition for preterm infants after discharge. Arch Dis Child. 1992;67(3):324-7.

28. Lucas A, Fewtrell MS, Morley R, Lucas PJ, Baker BA, Lister G, et al. Randomized outcome trial of human milk fortification and developmental outcome in preterm infants. Am J Clin Nutr. 1996;64(2):142-51.

29. Lucas A, Fewtrell MS, Morley R, Singhal A, Abbott RA, Isaacs E, et al. Randomized trial of nutrient-enriched formula versus standard formula for postdischarge preterm infants. Pediatrics. 2001;108(3):703-11.

30. Moltu SJ, Strommen K, Blakstad EW, Almaas AN, Westerberg AC, Braekke K, et al. Enhanced feeding in very-low-birth-weight infants may cause electrolyte disturbances and septicemia--a randomized, controlled trial. Clin Nutr. 2013;32(2):207-12.

31. Mukhopadhyay K, Narnag A, Mahajan R. Effect of human milk fortification in appropriate for gestation and small for gestation preterm babies: a randomized controlled trial. Indian Pediatr. 2007;44(4):286-90.

32. O'Connor DL, Khan S, Weishuhn K, Vaughan J, Jefferies A, Campbell DM, et al. Growth and nutrient intakes of human milk-fed preterm infants provided with extra energy and nutrients after hospital discharge. Pediatrics. 2008;121(4):766-76.

33. Peng CC, Hsu CH, Kao HA, Hung HY, Chang JH. Feeding with premature or infant formula in premature infants after discharge: comparison of growth and nutrition status. Acta Paediatr Taiwan. 2004;45(3):151-7.

34. Pettifor JM, Rajah R, Venter A, Moodley GP, Opperman L, Cavaleros M, et al. Bone mineralization and mineral homeostasis in very low-birth-weight infants fed either human milk or fortified human milk. J Pediatr Gastroenterol Nutr. 1989;8(2):217-24.

35. Picaud JC, Decullier E, Plan O, Pidoux O, Bin-Dorel S, van Egroo LD, et al. Growth and bone mineralization in preterm infants fed preterm formula or standard term formula after discharge. J Pediatr. 2008;153(5):616-21, 21 e1-2.

36. Roggero P, Gianni ML, Amato O, Liotto N, Morlacchi L, Orsi A, et al. Growth and fat-free mass gain in preterm infants after discharge: a randomized controlled trial. Pediatrics. 2012;130(5):e1215-21.

37. Svenningsen NW, Lindroth M, Lindquist B. A comparative study of varying protein intake in low birthweight infant feeding. Acta Paediatr Suppl. 1982;296:28-31.

38. Tan MJ, Cooke RW. Improving head growth in very preterm infants--a randomised controlled trial I: neonatal outcomes. Arch Dis Child Fetal Neonatal Ed. 2008;93(5):F337-41.

39. Yu MX, Zhuang SQ, Gao XY, Tong XM, Yue SJ, Shi LP, et al. Effects of a nutrient-dense formula compared with a post-discharge formula on post-discharge growth of preterm very low birth weight infants with extrauterine growth retardation: a multicentre randomised study in China. J Hum Nutr Diet. 2020.

40. Wauben IP, Atkinson SA, Shah JK, Paes B. Growth and body composition of preterm infants: influence of nutrient fortification of mother's milk in hospital and breastfeeding post-hospital discharge. Acta Paediatr. 1998;87(7):780-5.

41. Wheeler RE, Hall RT. Feeding of premature infant formula after hospital discharge of infants weighing less than 1800 grams at birth. J Perinatol. 1996;16(2 Pt 1):111-6.

42. Zachariassen G, Faerk J, Grytter C, Esberg BH, Hjelmborg J, Mortensen S, et al. Nutrient enrichment of mother's milk and growth of very preterm infants after hospital discharge. Pediatrics. 2011;127(4):e995-e1003.
