# Supplementary material for: Impact of macronutrient supplements on later growth of children born preterm or small for gestational age: A systematic review and meta-analysis of randomised and quasirandomised controlled trials
Source: PLoS Med. 2020 May 26;17(5):e1003122. doi: 10.1371/journal.pmed.1003122 (PMC7250404; doi:10.1371/journal.pmed.1003122)
Supplement: S3 Table — (DOCX) [file pmed.1003122.s005.docx]

**S3 Table. Macronutrient intakes between trials using formula as primary feed and breast milk as primary feed**

|  | **Formula as primary feed** | | **Breast milk as primary feed** | | P Value |
| --- | --- | --- | --- | --- | --- |
|  | Mean | SD | Mean | SD |  |
| **Mean intakes in the supplemented groups** | | | | | |
| Protein (g/100ml) | 2.03 | 0.23 | 2.01 | 0.23 | 0.86 |
| Fat (g/100ml) | 4.12 | 0.46 | 3.97 | 0.44 | 0.54 |
| Carbohydrate (g/100ml) | 7.86 | 1.00 | 9.11 | 1.35 | 0.03 |
| Energy (g/100ml) | 75.76 | 7.59 | 80.24 | 3.33 | 0.22 |
| **Mean intake in the unsupplemented groups** | | | | | |
| Protein (g/100ml) | 1.53 | 0.20 | 1.36 | 0.15 | 0.06 |
| Fat (g/100ml) | 3.70 | 0.27 | 4.23 | 0.08 | <0.001 |
| Carbohydrate (g/100ml) | 7.25 | 0.26 | 7.34 | 0.91 | 0.80 |
| Energy (g/100ml) | 68.19 | 3.20 | 68.86 | 4.91 | 0.68 |
| **Mean differences intakes between supplemented and unsupplemented groups** | | | | | |
| Protein (g/100ml) | 0.49 | 0.16 | 0.64 | 0.18 | 0.06 |
| Fat (g/100ml) | 0.42 | 0.43 | -0.27 | 0.42 | 0.004 |
| Carbohydrate (g/100ml) | 0.64 | 0.98 | 1.63 | 1.08 | 0.06 |
| Energy (g/100ml) | 7.57 | 7.14 | 10.64 | 4.38 | 0.37 |

The composition information for formulae were extracted from the publications and the composition of breastmilk was estimated according to the recent guideline [1].

Reference

1. National Health & Medical Research Council (NHMRC). Dietary guidelines for children and aolescents in Australia - incorporating the infant feeding guidelines for health workers. Australia: The National Health and Medical Research Council; 2003 [updated 10 April 2003; cited 2019 17 June ]. Available from: <http://childaustralia.mrooms.net/pluginfile.php/4134/mod_page/content/38/diet-guidelines.pdf>
