# Supplementary material for: Impact of macronutrient supplements on later growth of children born preterm or small for gestational age: A systematic review and meta-analysis of randomised and quasirandomised controlled trials
Source: PLoS Med. 2020 May 26;17(5):e1003122. doi: 10.1371/journal.pmed.1003122 (PMC7250404; doi:10.1371/journal.pmed.1003122)
Supplement: S4 Table — (DOCX) [file pmed.1003122.s006.docx]

**S4 Table. Macronutrient intakes between trials conducted up to 2000 and those conducted after 2000**

| **Table 3. Estimated intakes of protein, fat, carbohydrate and energy in trials conducted up to 2000 and those conducted after 2000.** | | | | | |
| --- | --- | --- | --- | --- | --- |
|  | Before and during 2000 | | After 2000 | | P Value |
|  | Mean | SD | Mean | SD |  |
| **Mean intakes in the unsupplemented groups** | | | | | |
| Protein (g/100ml) | 1.43 | 0.09 | 1.56 | 0.27 | 0.11 |
| Fat (g/100ml) | 3.84 | 0.26 | 3.85 | 0.41 | 0.97 |
| Carbohydrate (g/100ml) | 7.10 | 0.16 | 7.47 | 0.68 | 0.10 |
| Energy (g/100ml) | 67.27 | 0.96 | 69.62 | 5.01 | 0.12 |
| **Mean differences intakes between supplemented and unsupplemented groups** | | | | | |
| Protein (g/100ml) | 0.51 | 0.17 | 0.54 | 0.18 | 0.65 |
| Fat (g/100ml) | 0.26 | 0.42 | 0.28 | 0.60 | 0.93 |
| Carbohydrate (g/100ml) | 0.75 | 1.22 | 0.94 | 0.94 | 0.68 |
| Energy (g/100ml) | 7.54 | 6.84 | 8.78 | 4.65 | 0.65 |

The composition information for formulae were extracted from the publications and the estimated composition of breastmilk was according to the recent guideline [1].

Reference

1. National Health & Medical Research Council (NHMRC). Dietary guidelines for children and aolescents in Australia - incorporating the infant feeding guidelines for health workers. Australia: The National Health and Medical Research Council; 2003 [updated 10 April 2003; cited 2019 17 June ]. Available from: <http://childaustralia.mrooms.net/pluginfile.php/4134/mod_page/content/38/diet-guidelines.pdf>
