# Supplementary figures and images for: Impact of macronutrient supplements on later growth of children born preterm or small for gestational age: A systematic review and meta-analysis of randomised and quasirandomised controlled trials
Source: PLoS Med. 2020 May 26;17(5):e1003122. doi: 10.1371/journal.pmed.1003122 (PMC7250404; doi:10.1371/journal.pmed.1003122)

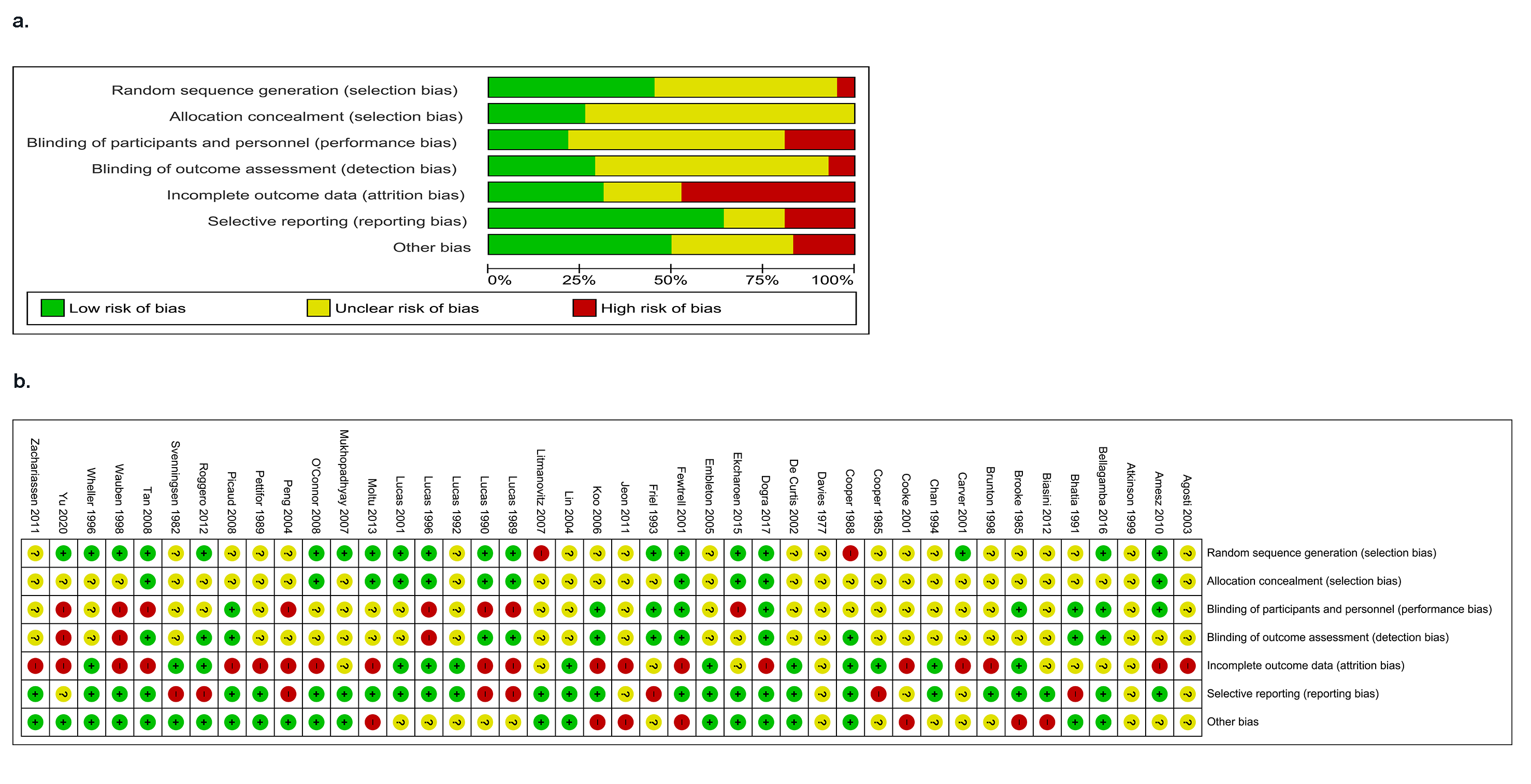

Supplement: S1 Fig — (a) Risk of bias graph: review authors’ judgements about each risk of bias item presented as percentages across all included studies. (b) Risk bias summary: review authors’ judgements about each risk of bias item for each included study. (TIF) [file pmed.1003122.s007.tif]

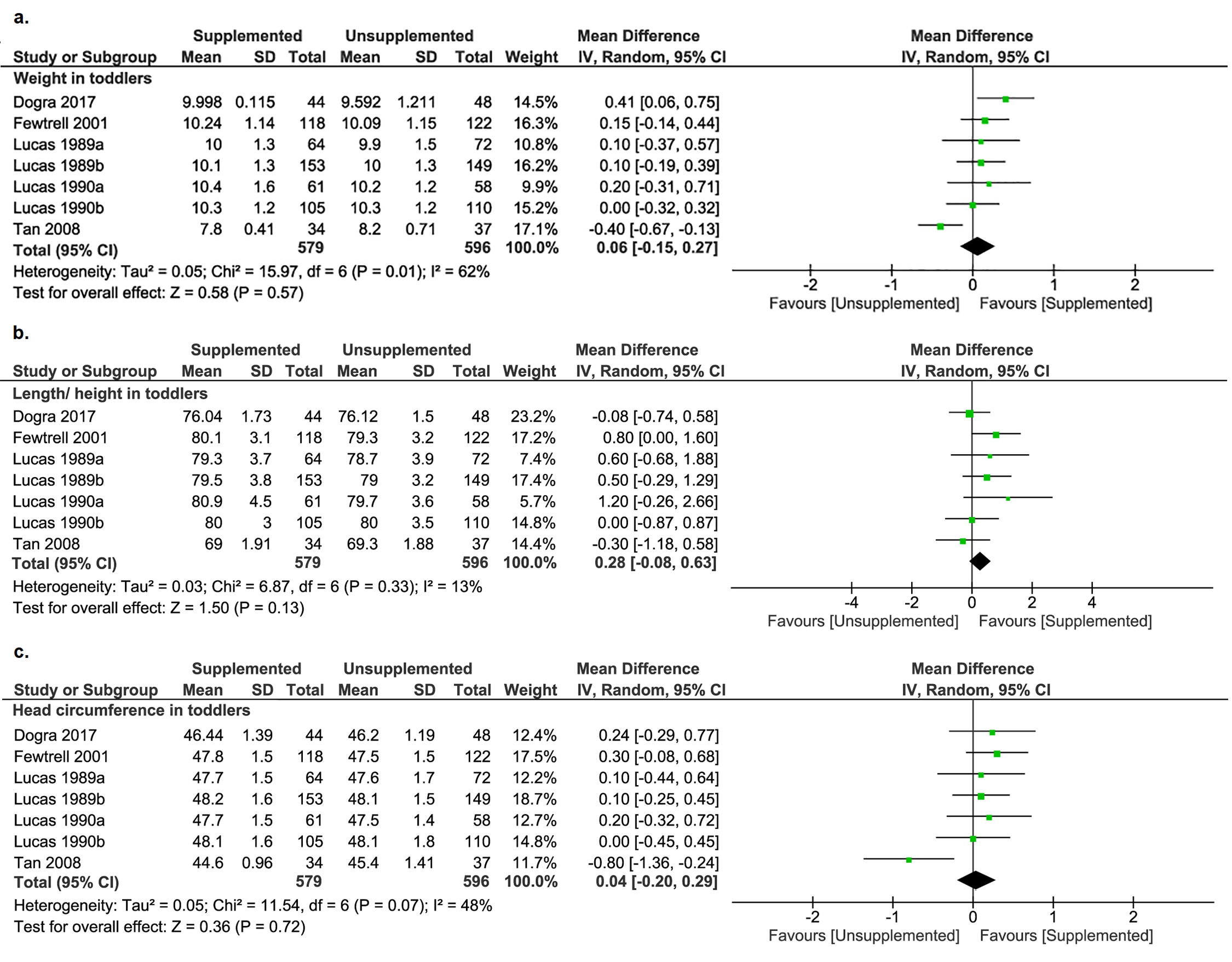

Supplement: S2 Fig — Forest plots of effect of macronutrient supplementation on growth outcomes including trials with low risk of bias. P-values are from Z test for the summary effect and chi-squared test for heterogeneity. (a) Weight in toddlers (kg), (b) length or height in toddlers (cm), (c) head circumference in toddlers (cm). (TIF) [file pmed.1003122.s008.tif]

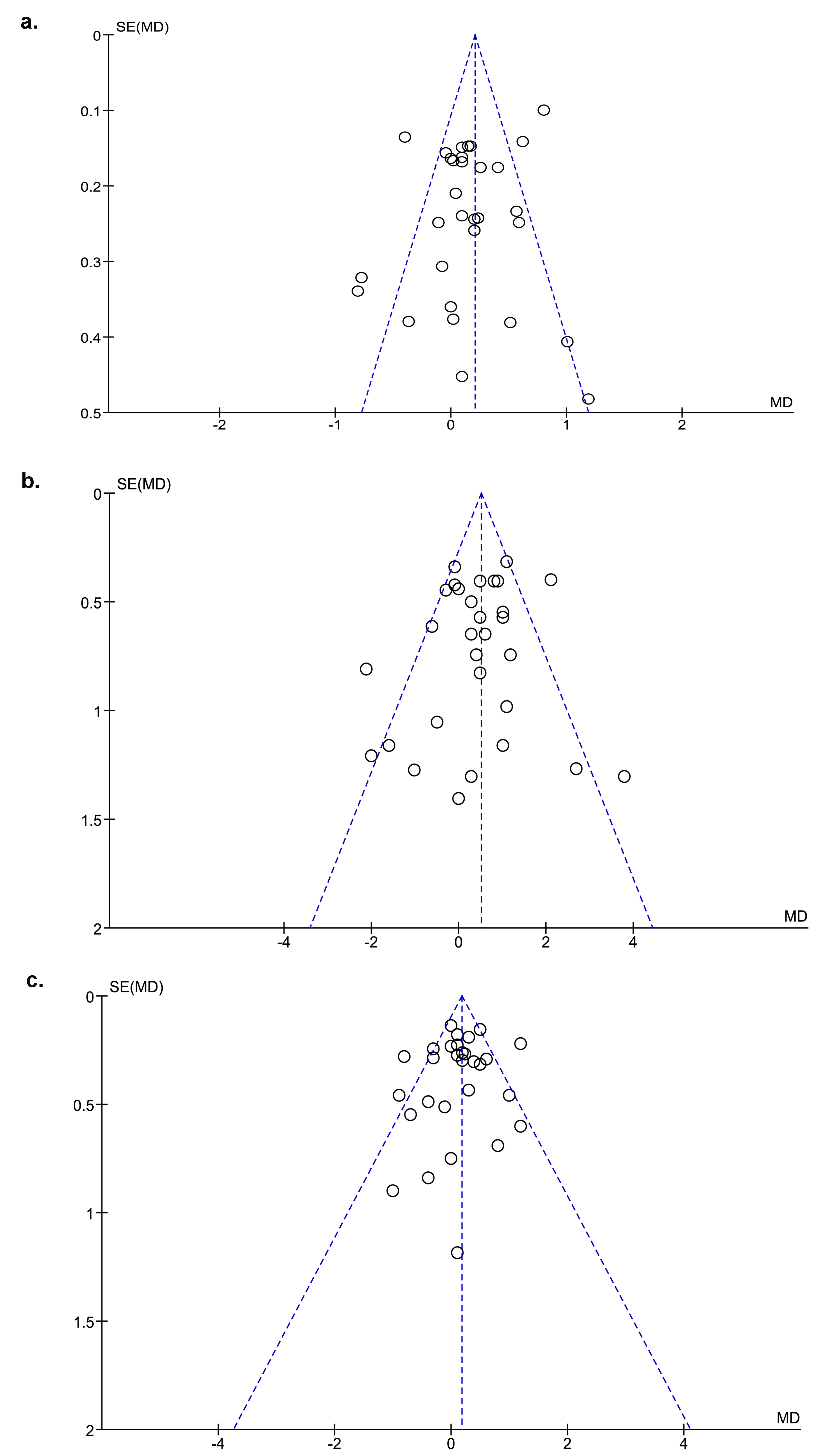

Supplement: S3 Fig — Funnel plot of supplemented versus unsupplemented nutrition for the growth outcomes. (a) Weight in toddlers, (b) length or height in toddlers, (c) head circumference in toddlers. The middle dashed line indicates the overall MD. The dashed line either side represents the pseudo-95% CIs. CI, confidence interval; MD, mean difference. (TIF) [file pmed.1003122.s009.tif]

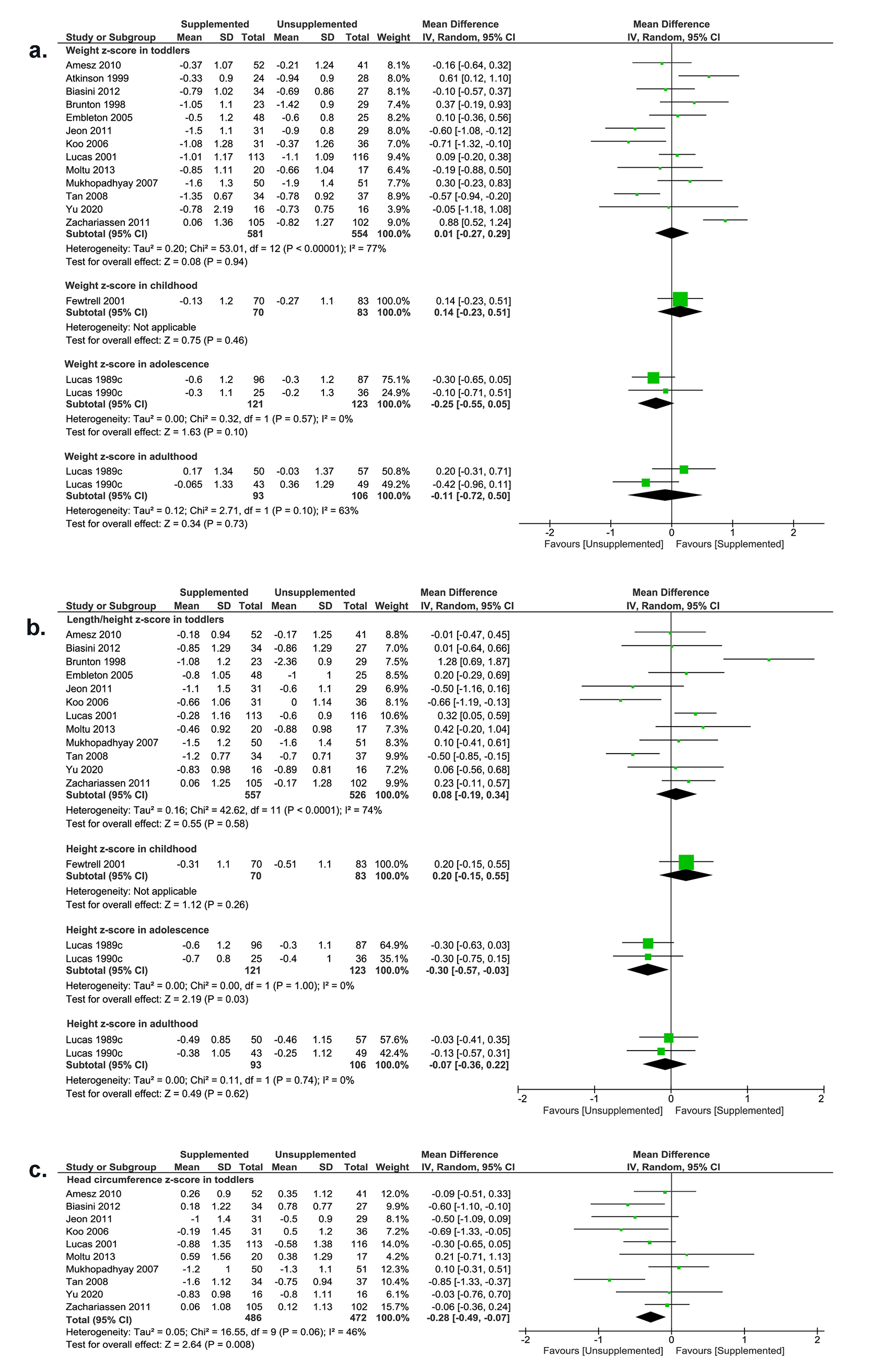

Supplement: S4 Fig — P-values are from Z test for the summary effect and chi-squared test for heterogeneity. (a) Weight z-scores, (b) length/ height z-scores, (c) head circumference z-scores. (TIF) [file pmed.1003122.s010.tif]

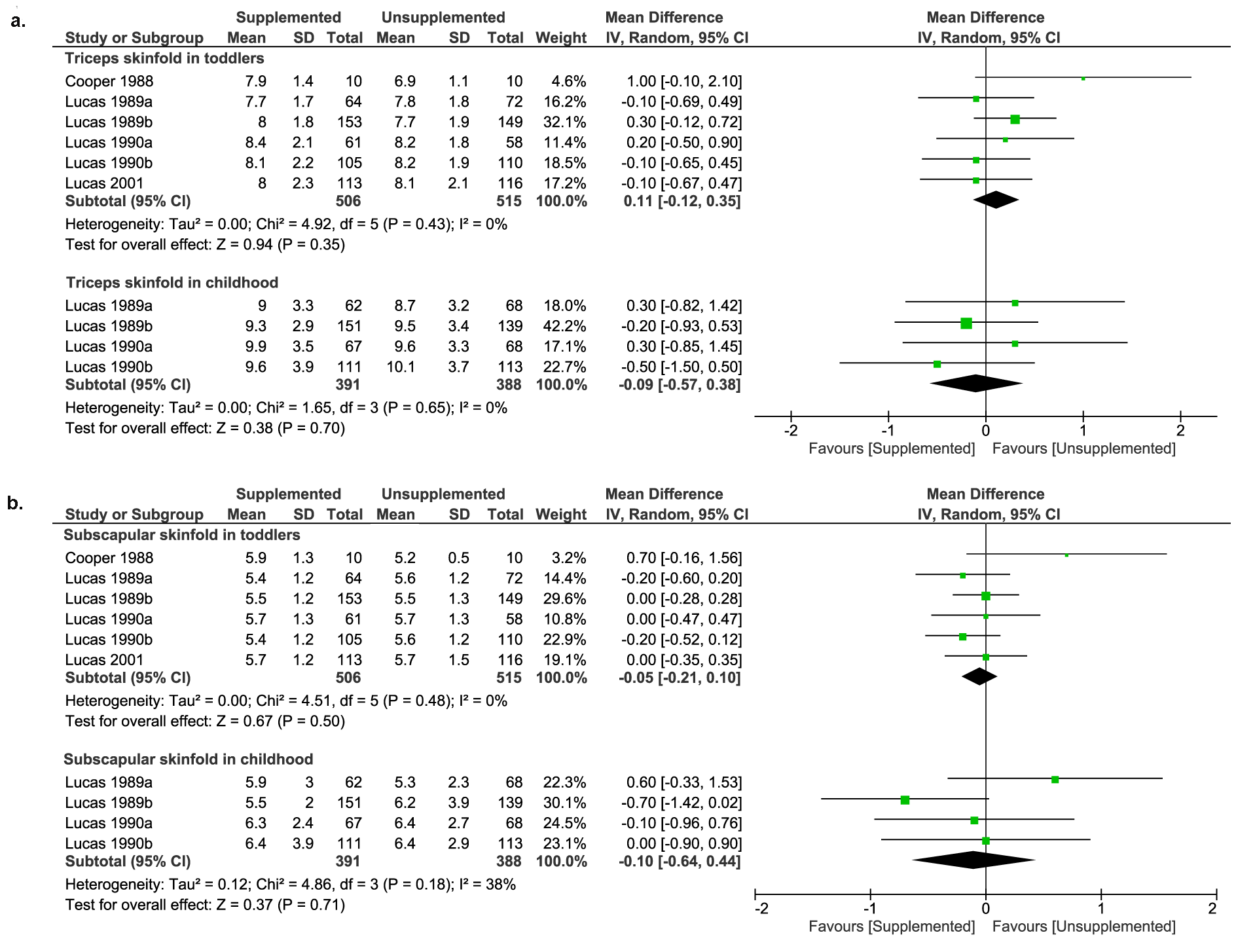

Supplement: S5 Fig — P-values are from Z test for the summary effect and chi-squared test for heterogeneity. (a) Triceps skin fold thickness (mm), (b) subscapular skin fold thickness (mm). (TIF) [file pmed.1003122.s011.tif]

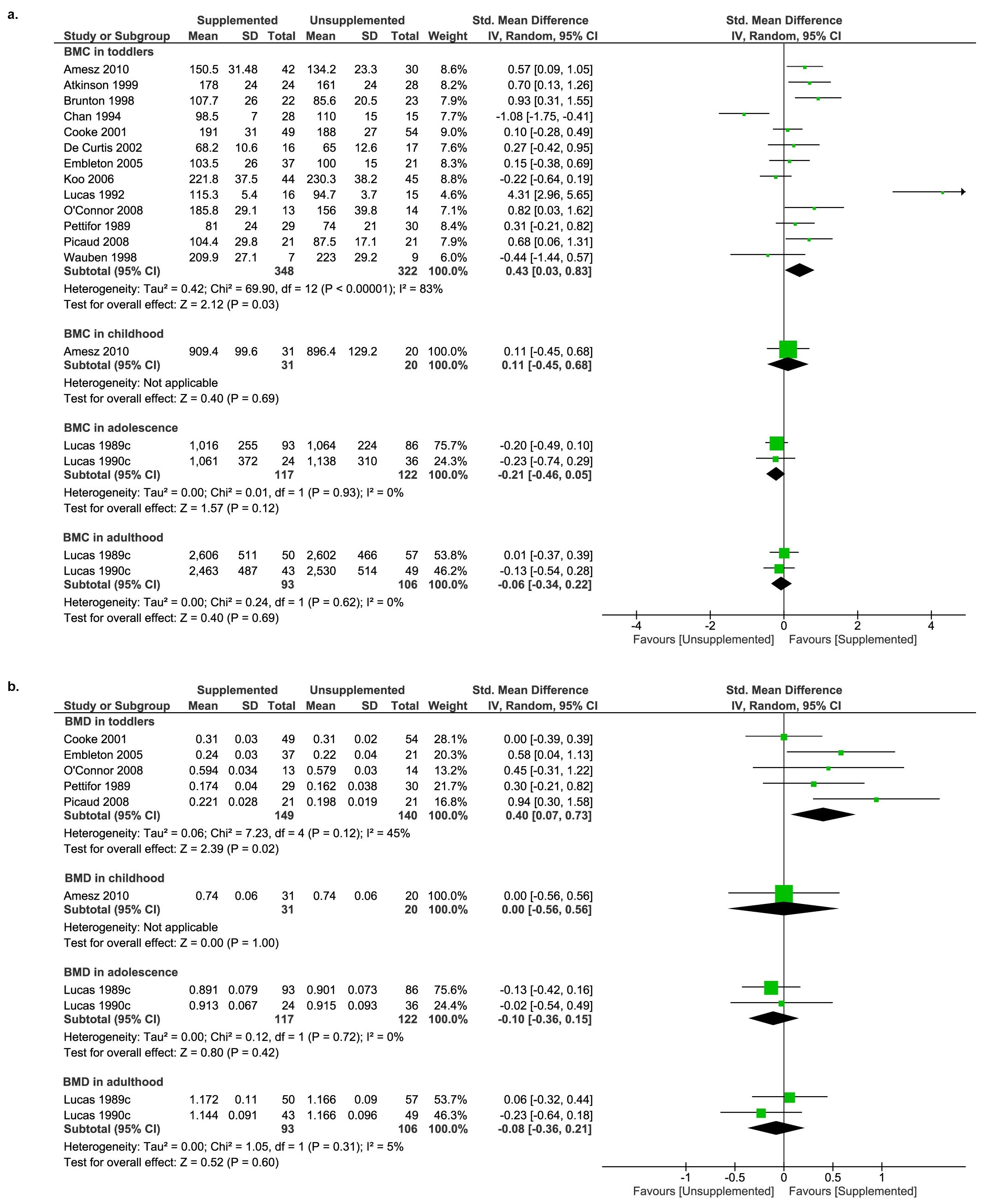

Supplement: S6 Fig — P-values are from Z test for the summary effect and chi-squared test for heterogeneity. Std.Mean Difference = difference in mean outcome between groups/standard deviation of outcome among participants. (a) BMC, (b) BMD. BMC, bone mineral content; BMD, bone mineral density. (TIF) [file pmed.1003122.s012.tif]
