# Supplementary material for: Impact of macronutrient supplements on later growth of children born preterm or small for gestational age: A systematic review and meta-analysis of randomised and quasirandomised controlled trials
Source: PLoS Med. 2020 May 26;17(5):e1003122. doi: 10.1371/journal.pmed.1003122 (PMC7250404; doi:10.1371/journal.pmed.1003122)
Supplement: S1 Protocol — SGA, small for gestational age. (DOCX) [file pmed.1003122.s014.docx]

**Impact of macronutrient supplements on later growth of children born preterm or small for gestational age: Protocol for a systematic review and meta-analysis**

**Authors**

Luling Lin^1^;

Emma Amissah^1^;

Gregory D. Gamble^1^;

Caroline A. Crowther^1^;

Jane E. Harding^1`^;

**Affiliations**

^1^Liggins Institute, University of Auckland, Auckland, New Zealand

**Background**

Infants born alive at less than 37 completed weeks of gestation have been defined as preterm [1]. About 15 million infants are born preterm around the world annually and the rates of preterm birth are increasing in most countries [2]. Preterm birth accounts for 3.1% of all Disability Adjusted Life Years [3]. Small for gestational age (SGA) infants are those smaller in size than normal for their gestational age, and most commonly defined as a weight below the 10th percentile for the gestational age [4]. Preterm and SGA infants are both at increased risk of poor growth, slow development and disability [2,5-7] As adults, they are at increased risk of obesity, diabetes, and cardiovascular disease [8].

Most preterm and SGA infants attain "catch-up" growth through childhood to adolescent and into adulthood [9]. However, failure to “catch-up” is very common among these population due to the inadequate protein and energy intake [10-12]. Most preterm infants accumulate significant nutrient deficits during their early life [10] and they are considerably smaller than their full-term peers. Further, preterm infants often have significantly lower bone mineral content than those born at term, and the prevalence of metabolic bone disease is inversely associated with birth weight and gestational age [13-15]. Metabolic bone disease during infancy in turn may increase the risk of neonatal rickets, childhood fracture, and poor growth. Metabolic bone disease during infancy in turn may increase the risk of neonatal rickets, childhood fracture, and poor growth [15]. Providing preterm and SGA infants with enhanced nutrition in their early life may benefit short and long term growth.

**Objective**

To assess effects of macronutrient supplements in nutrition of preterm and small-for-gestational-age infants on growth and bone outcomes after hospital discharge.

**Methods**

**Criteria for considering studies for this review.**

**Type of studies**

Randomised controlled trials (RCTs) and quasi-RCTs without restrictions on date of publication or language.

**Types of participants**

Infants born preterm (< 37 weeks’ gestation) or born small (birthweight <2.5 kg or <10th centile for gestational age).

**Types of intervention and comparison**

Studies will be included if the intervention was intended to increase the intake of one or more macronutrients (protein, carbohydrate, fat, energy content, or protein to energy ratio) with the primary aim of improving growth and development of the infant. Interventions can be enteral or parenteral or a combination of enteral and parenteral supplements and commence any time during initial hospitalisation after birth or after discharge from hospital, and must be provided for a minimum duration of one week. Outcome data must be reported beyond term equivalent age (> 37 weeks’ gestation) or following discharge from hospital after birth.

Studies that report on comparisons between unsupplemented nutrition and supplemented nutrition with parental supplements, human breast milk supplements, formula milk or other macronutrients will be eligible for inclusion.

Trials reporting the following comparisons will be eligible for inclusion:

- Parenteral formulation A versus parenteral formulation B with different macronutrient composition.
- Human milk (mother’s own or donor) versus supplemented human milk (mother’s own or donor).
- Human milk (mother’s own or donor) versus formula milk (term or preterm).
- Supplemented human milk (mother’s own or donor) versus formula (term or preterm) of different macronutrient composition.
- Supplemented human milk A (mother’s own or donor) versus supplemented human milk B (mother’s own or donor).
- Formula A versus Formula B with different macronutrient composition (including preterm versus term formula, Brand A versus Brand B).

We will exclude trials that examine the timing of the introduction of nutrition (early versus delayed feeding), that compare macronutrients of different composition (e.g. different types of lipids or proteins); studies whose outcomes focus on gastrointestinal development rather than growth and development, and studies reporting on variations in composition of micronutrients (including sodium, potassium, calcium, phosphorous, vitamins, other minerals, amino acids, fatty acids).

**Types of outcome measures**

Outcomes have been selected to reflect the most clinically important measures of safety and effectiveness for this population group.

Outcomes:

The outcomes will be evaluated in toddlers (less than 3 years), childhood (3 to 8 years), adolescence (9 to 18 years) and adulthood ( >18 years).

Primary outcome: Body mass index (BMI) in childhood (3 to 8 years).

Secondary outcomes**:**

- Growth assessments
- Weight (raw data and z scores)
- Length or height (raw data and z scores)
- Head circumference (raw data and z scores)
- Ponderal Index
- Body mass index (BMI)
- Body composition (fat mass, fat free mass, measured by bioimpedance or DEXA or skin fold thickness or other method)
- Bone development
- Bone mineral content
- Volumetric bone mineral density
- Bone fractures
- Nutrition
- Feeding tolerance
- Intake (protein, energy)
- Appetite
- Breast feeding and duration
- Death: neonatal or later death, up to the time of follow-up and cause of death
- Quality of life
- General health and use of healthcare resources.
- Adverse events.
- Cost

## **Search methods for identification of studies**

**Search strategy：**

An independent information specialist will develop a search strategy that will be used to search the following electronic databases.

- Medline
- Embase
- CENTRAL
- Cochrane Database of Systematic Reviews

We will also search for registered trials in:

- Current Controlled Trials ([www.controlled-trials.com](http://www.controlled-trials.com))
- Clinical Trials ([www.clinicaltrials.gov](http://www.clinicaltrials.gov))
- Australian and New Zealand Clinical Trials Registry ([www.anzctr.org.au](http://www.anzctr.org.au))

Experts in the field and trialists will be asked if they can identify other published or ongoing trials. Potentially eligible trials that are not yet completed will not be included in this systematic review but will be noted for inclusion in future updates. Conference abstracts will be included if they have usable summary data.

**Data collection and analysis**

**Selection of studies**

Two review authors will screen the titles and abstracts of all references identified by the above search strategy independently. Any discrepancies will be resolved by discussion or by a third review author. We will obtain the full text of all potentially relevant articles, and link the articles from the same study together. We will assess full-text articles for inclusion or exclusion using the eligibility criteria independently. Any conflicts will be resolved by discussion or by a third author. Investigators will be corresponded with as needed to clarify study eligibility and obtain missing results if possible.

**Data extraction and management**

We will extract data into a template data extraction form for each included study. The following information will be extracted: study setting, study methodology, information for assessment of the risk of bias, participant characteristics, intervention and control details and outcome data. Conflicts in the data extraction process will be resolved by discussion or by a third author.

**Assessment of risk of bias in included studies**

Two review authors will independently assess the risk of bias for each study using the criteria outlined in the Cochrane Handbook for Systematic Reviews of Interventions [16]. We will evaluate and record the risk of bias as high, low, or unclear across the following domains and present our findings in a 'Risk of bias' table and summary figure.

**Random sequence generation (checking for possible selection bias)**

We will describe for each included study the method used to generate the allocation sequence in sufficient detail to allow an assessment of whether it should produce comparable groups.

We will assess the method as:

• Low risk of bias (any truly random process, e.g. random number table; computer random number generator; coin tossing).

• High risk of bias (any non-random process, e.g. odd or even; date of admission; hospital or clinic record number).

• Unclear risk of bias (Insufficient information about the sequence generation process to permit judgement of 'Low risk' or 'High risk').

**Allocation concealment (checking for possible selection bias)**

We will describe for each included study the method used to conceal allocation to interventions prior to assignment and assess whether intervention allocation could have been foreseen in advance of, or during recruitment, or changed after assignment.

We will assess the methods as:

• Low risk of bias (e.g. telephone or central randomisation; consecutively numbered sealed opaque envelopes, sequentially numbered drug containers of identical appearance).

• High risk of bias (open random allocation; unsealed or non-opaque envelopes, alternation; date of birth).

• Unclear risk of bias (Insufficient information to permit judgement of 'Low risk' or 'High risk').

### Blinding of participants and personnel (checking for possible performance bias)

We will describe for each included study the methods used, if any, to blind study participants and personnel from which intervention a participant received. We will consider studies to be at low risk of bias if they were blinded, or if we judge that the lack of blinding would be unlikely to affect results.

We will assess the methods as:

• Low risk of bias (e.g. no blinding or incomplete blinding, but the review authors judge that the outcome is not likely to be influenced by lack of blinding; blinding of participants and key study personnel ensured, and unlikely that the blinding could have been broken).

• High risk of bias (e.g. no blinding or incomplete blinding, and the outcome is likely to be influenced by lack of blinding; blinding of key study participants and personnel attempted, but likely that the blinding could have been broken, and the outcome is likely to be influenced by lack of blinding).

• Unclear risk of bias (e.g. insufficient information to permit judgement of 'Low risk' or 'High risk'; the study did not address this outcome.)

### Blinding of outcome assessment (checking for possible detection bias)

We will describe for each included study the methods used, if any, to blind outcome assessors from knowledge of which intervention participant received.

We will assess the methods used to blind outcome assessment as

• Low risk of bias (e.g. no blinding of outcome assessment, but the review authors judge that the outcome measurement is not likely to be influenced by lack of blinding; blinding of outcome assessment ensured, and unlikely that the blinding could have been broken).

• High risk of bias (e.g. no blinding of outcome assessment, and the outcome measurement is likely to be influenced by lack of blinding; blinding of outcome assessment, but likely that the blinding could have been broken, and the outcome measurement is likely to be influenced by lack of blinding.).

• Unclear risk of bias (e.g. insufficient information to permit judgement of 'Low risk' or 'High risk'; the study did not address this outcome.)

**Incomplete outcome data (checking for possible attrition bias due to the amount, nature and handling of incomplete outcome data)**

We will describe for each included study the completeness of data including attrition and exclusions from the analysis. We will state whether attrition and exclusions are reported and the numbers included in the analysis at each stage (compared with the total number of randomised participants, and numbers eligible for each study phase), reasons for attrition or exclusions where reported, and whether missing data are balanced across groups or are related to outcomes. Where sufficient information is reported, we will re-include missing data in the analyses that are undertaken.

We will assess the methods as:

• Low risk of bias (e.g. no missing outcome data; missing outcome data balanced across groups);

• High risk of bias (e.g. numbers or reasons for missing data imbalanced across groups; ‘as treated’ analysis done with substantial departure of intervention received from that assigned at randomisation);

• Unclear risk of bias.

**Measures of treatment effect**

**Dichotomous data**

We will use the numbers of events in the control and intervention groups of each study to calculate risk ratios (RRs) with 95% confidence intervals (CIs) for dichotomous data.

**Continuous data**

We will calculate mean differences (MDs) with 95% CIs between treatment groups where outcomes are measured in the same way for continuous data. Standardised mean differences (SMDs) will be used if the outcomes from trials are the same but different methods have been used to collect the data.

**Unit of analysis issues**

If we identify cluster-randomised trials, we will undertake analysis at the individual level taking clustering into account as described in the Cochrane Handbook for Systematic Reviews of Interventions [16].

**Assessment of heterogeneity**

We will consider whether the clinical and methodological characteristics of the included studies are sufficiently similar for meta-analysis to provide a clinically meaningful summary. We calculated the *I*² statistic and *Chi*^2^ for each analysis to quantify inconsistency across studies and describe the percentage of variability in effect estimates that may be due to heterogeneity rather than sampling error. In the event that we observe substantial heterogeneity (I² > 50% and P value< 0.10 in the Chi^2^ test), we will explore the possible causes (for example, differences in study design, participants, interventions, or completeness of outcome assessments) in sensitivity analyses.

**Assessment of reporting biases**

We will assess publication bias by visual inspection of a funnel plot, plotting the study effect size against the sample size, if there are enough studies (10 or more trials) to make such an inspection valid. Two authors will examine the methods of each study for primary outcomes. If asymmetry is apparent, we will consider and discuss possible reasons for it.

**Data synthesis**

We will use a random-effect meta-analysis for combining data where it is reasonable to assume that studies were estimating the same underlying treatment effect. If there is evidence of clinical heterogeneity sufficient to expect that the underlying treatment effects differ between trials, or if substantial statistical heterogeneity is detected, we will undertake sensitivity and/or subgroup analyses to attempt to explain the heterogeneity for primary outcomes.

##

**Quality of evidence**

We will use the Grading of Recommendations Assessment, Development and Evaluation (GRADE), as outlined in the GRADE Handbook 2013, to assess the quality of evidence for the following outcomes: cognitive impairment and metabolic risk after hospital discharge.

We will assess the quality of evidence for the main comparison at the outcome level using the GRADE approach[17]. We will consider evidence from randomised controlled trials as high quality that may be downgraded on the basis of consideration of any of five areas.

- Design (risk of bias)
- Consistency across studies
- Directness of the evidence
- Precision of estimates
- Presence of publication bias

We will use the **GradePro GDT** Guideline Development Tool (https://gradepro.org/) to create a ‘Summary of findings’ table to report the quality of the evidence for the following outcomes:

1. BMI at >3 years

2. Height at >3 years

3. Lean mass at >3 years

4. Fat mass at >3 years

5. Weight in toddlers

6. Head circumference in toddlers

7. Length in toddlers

The GRADE approach results in assessment of the quality of a body of evidence according to four grades[17]:

- High: We are very confident that the true effect lies close to the estimate of effect.
- Moderate: We are moderately confident in the effect estimate: The true effect is likely to be close to the estimate of effect but may be substantially different.
- Low: Our confidence in the effect estimate is limited: The true effect may be substantially different from the estimate of effect.
- Very low: We have very little confidence in the effect estimate: The true effect is likely to be substantially different from the estimate of effect.

**Subgroup analysis**

If substantial heterogeneity is identified, subgroup and sensitivity analyses will be performed. We will consider whether an overall summary is meaningful and if it is, we will use a random effects model to analyse it.

We plan to carry out the following subgroup analyses to evaluate differences in primary outcomes between:

1. Sex of infant (male vs female);
2. Size of infant at birth (1kg or less vs more than 1kg at birth);
3. Size for gestation of the infant (10th centile or less vs more than 10th centile);
4. Gestational age of infant at birth (28 completed weeks or less vs 29 to 32 completed weeks vs 33 to 36 weeks)
5. Timing of supplement

In hospital nutrition: the intervention was commenced in hospital or on average ended at 42 weeks’ postmenstrual age or earlier,

Post-discharge nutrition: the intervention was commenced after discharge or on average started at 36 weeks’ postmenstrual age or later,

Both in hospital and post-discharge nutrition: the intervention was commenced in the hospital and continued post-discharge;

1. Type of supplement (protein vs carbohydrate vs fat vs multicomponent and their interactions);
2. Breast milk vs formula as primary milk feed;
3. Duration of supplement (1 to 2 weeks vs 3 to 6 weeks vs more than 7 weeks);
4. Different epochs (conducted up to the year of 2000 vs conducted in or after the year of 2001);

**Sensitivity analysis**

## If sufficient data are available, we will conduct sensitivity analysis by examining only those trials considered to have a low risk of bias for selection and detection bias.

**Reference**

1. World Health Organization. Preterm birth 2016; World Health Organization. Available from: <http://www.who.int/mediacentre/factsheets/fs363/en/>

2. Blencowe H, Cousens S, Chou D, Oestergaard M, Say L, Moller AB, et al. Born too soon: the global epidemiology of 15 million preterm births. Reprod Health. 2013;10 Suppl 1:S2.

3. Valero De Bernabe J, Soriano T, Albaladejo R, Juarranz M, Calle ME, Martinez D, et al. Risk factors for low birth weight: a review. Eur J Obstet Gynecol Reprod Biol. 2004;116(1):3-15.

4. Clayton PE, Cianfarani S, Czernichow P, Johannsson G, Rapaport R, Rogol A. Management of the child born small for gestational age through to adulthood: A consensus statement of the international societies of pediatric endocrinology and the Growth Hormone Research Society. JCEM. 2007;92(3):804-10.

5. Scharf RJ, Stroustrup A, Conaway MR, DeBoer MD. Growth and development in children born very low birthweight. Arch Dis Child Fetal Neonatal Ed. 2016;101(5):F433-F8.

6. Katz J, Lee ACC, Kozuki N, Lawn JE, Cousens S, Blencowe H, et al. Mortality risk in preterm and small-for-gestational-age infants in low-income and middle-income countries: a pooled country analysis. Lancet. 2013;382(9890):417-25.

7. Christian P, Lee SE, Angel MD, Adair LS, Arifeen SE, Ashorn P, et al. Risk of childhood undernutrition related to small-for-gestational age and preterm birth in low- and middle-income countries. Int J Epidemiol. 2013;42(5):1340-55.

8. Mericq V, Martinez-Aguayo A, Uauy R, Iniguez G, Van der Steen M, Hokken-Koelega A. Long-term metabolic risk among children born premature or small for gestational age. Nat Rev Endocrinol. 2017;13(1):50-62.

9. Sullivan MC, McGrath MM, Hawes K, Lester BM. Growth trajectories of preterm infants: birth to 12 years. J Pediatr Health Care. 2008;22(2):83-93.

10. Embleton NE, Pang N, Cooke RJ. Postnatal malnutrition and growth retardation: an inevitable consequence of current recommendations in preterm infants? Pediatrics. 2001;107(2):270-3.

11. Hay WW, Jr. Nutritional requirements of extremely low birthweight infants. Acta Paediatr Suppl. 1994;402:94-9.

12. Cooke RW. Conventional birth weight standards obscure fetal growth restriction in preterm infants. Arch Dis Child Fetal Neonatal Ed. 2007;92(3):F189-92.

13. Rustico SE, Calabria AC, Garber SJ. Metabolic bone disease of prematurity. J Clin Transl Endocrinol. 2014;1(3):85-91.

14. Embleton N, Wood CL. Growth, bone health, and later outcomes in infants born preterm. J Pediatr (Rio J). 2014;90(6):529-32.

15. Rehman MU, Narchi H. Metabolic bone disease in the preterm infant: current state and future directions. World J Methodol. 2015;5(3):115-21.

16. Higgins JPT, Green S (editors), The Cochrane Collaboration. Cochrane handbook for systematic reviews of interventions version 5.1.0 [updated March 2011] 2011 [cited 2019 Mar 8]. Available from: <www.cochrane-handbook.org>

17. Schünemann H, Brozek J, Guyatt G, Oxman A, editors. GRADE handbook for grading quality of evidence and strength of recommendations. Updated October 2013. 2013. Available from: guidelinedevelopment.org/handbook
